# Oral *Staphylococcus* Species and MRSA Strains in Patients with Orofacial Clefts Undergoing Surgical Rehabilitation Diagnosed by MALDI-TOF MS

**DOI:** 10.3390/pathogens13090763

**Published:** 2024-09-05

**Authors:** Mateus Cardoso Oliveira, Marcelo Fabiano Gomes Boriollo, Angélica Cristina de Souza, Thaísla Andrielle da Silva, Jeferson Júnior da Silva, Karina Teixeira Magalhães-Guedes, Carlos Tadeu dos Santos Dias, Wagner Luís de Carvalho Bernardo, José Francisco Höfling, Cristina Paiva de Sousa

**Affiliations:** 1 Department of Oral Diagnosis, Piracicaba Dental School, University of Campinas (FOP/UNICAMP), 901 Limeira Ave, Piracicaba 13414-903, SP, Brazil; mateus_oc1@hotmail.com (M.C.O.); thaislabiomed@hotmail.com (T.A.d.S.); jefersonbiomed@hotmail.com (J.J.d.S.); bernardowlc@gmail.com (W.L.d.C.B.); hofling2@unicamp.br (J.F.H.); 2 Center for Nursing and Health, State University of Southwest Bahia (UESB), José Moreira Sobrinho Avenue, Jequié 45205-490, BA, Brazil; 3 Department of Morphology and Pathology & Biotechnology Graduate Program (PPGBiotec), Center for Biological and Health Sciences (CCBS), Federal University of São Carlos (UFSCar), Km 235 Washington Luís Road, São Carlos 13565-905, SP, Brazil; prokarya@ufscar.br; 4 Department of Biology, Federal University of Lavras (UFLA), s/n Edmir Sá Santos Rotary Interchange, Lavras 37203-202, MG, Brazil; angelicacsouza.acs@gmail.com; 5 Department of Bromatological Analysis, Pharmacy Faculty, Federal University of Bahia (UFBA), 147 Barão de Jeremoabo Street, Salvador 40170-115, BA, Brazil; 6 Department of Exact Sciences, College of Agriculture, University of São Paulo (ESALQ/USP), 11 Pádua Dias Ave, Piracicaba 13418-900, SP, Brazil; ctsdias@usp.br

**Keywords:** MALDI-TOF MS, methicillin-resistant *Staphylococcus aureus*, orofacial cleft, pediatric patient, *Staphylococcus* spp.

## Abstract

This study investigated the occurrence and dynamics of oral *Staphylococcus* species in patients with orofacial clefts undergoing surgical rehabilitation treatment. Patients (*n* = 59) were statistically stratified and analyzed (age, gender, types of orofacial clefts, surgical history, and types of previous surgical rehabilitation). Salivary samples were obtained between hospitalization and the return to the specialized medical center. Microbiological diagnosis was performed by classical methods, and MALDI-TOF MS. MRSA strains (SCC*mec* type II, III, and IV) were characterized by the Decision Tree method. A total of 33 (55.9%) patients showed oral staphylococcal colonization in one, two, or three sampling steps. A high prevalence has been reported for *S. aureus* (including HA-, MRSA and CA-MRSA), followed by *S. saprophyticus*, *S. epidermidis*, *S. sciuri*, *S. haemolyticus*, *S. lentus*, *S. arlettae*, and *S. warneri*. The dynamics of oral colonization throughout surgical treatment and medical follow-up may be influenced by (i) imbalances in staphylococcal maintenance, (ii) efficiency of surgical asepsis or break of the aseptic chain, (iii) staphylococcal neocolonization in newly rehabilitated anatomical oral sites, and (iv) total or partial maintenance of staphylococcal species. The highly frequent clinical periodicity in specialized medical and dental centers may contribute to the acquisition of MRSA in these patients.

## 1. Introduction

The cleft lip palate (CLP) is by definition a malformation of congenital origin in the middle third of the face, with varying degrees of severity, beginning in the embryonic and early fetal stages. It is the more common head and neck deformity [1,2,3] of this origin, with a prevalence of 1.5 to 2 cases/1000 live births in several countries, being even more frequent in Brazil with 1/650 [4]. The exchanging that arises as a result of this malformation allows communication between the nasopharyngeal space and the oral cavity of patients with CLP, predisposing them to changes in the normal microbiota of both regions of the body [5,6,7]. These anatomical changes inevitably influence the composition of the oral and nasal microbiota, increasing the frequency of opportunistic pathogens, such as *Staphylococcus aureus* and other bacterial and fungal species [8,9,10,11,12,13,14,15].

Repeated hospitalizations and surgical interventions may negatively influence the oral microbiota of patients, especially in their first year of life, which may last until they are 20 years old [16,17]. Patients with CLP have several risk factors for opportunistic oral infectious diseases due to the anatomical changes in their maxillary segments, among others [8,14,18]. Infections, which can occur after primary surgery due to CLP, increase the risk of surgical wound rupture, palatine fistulas, and impaired growth of orofacial structures and speech, with the highest frequency of infections occurring by *S. aureus* or β-hemolytic *Streptococcus* [19]. In this context, *S. aureus* is one of the main causes of surgical wounds and infections associated with this condition, especially in the nasal membranes [20]. *S. aureus* can be found in nasal cavities and transmitted by oronasal fistula to the oral cavity, surviving in the oral environment of patients with CLP [21].

*S. aureus* infections are particularly problematic due to their frequent antibiotic resistance. Some strains of *S. aureus* can produce penicillinase, which hydrolyzes the β-lactam ring of penicillin and develops resistance to it. Later, scientists developed a new penicillinase-resistant semisynthetic penicillin called methicillin, which is resistant to β-lactamase hydrolysis. However, *S. aureus* has also shown a unique ability to respond rapidly to this new antibiotic by developing mechanisms for methicillin resistance and even for the newer antibiotics linezolid and daptomycin [22]. Studies have shown a positive relationship between fistulas, their sizes, and the presence of *S. aureus* [7]. In addition, recent studies [23,24] have shown the relevance of the oral cavity as a habitat of equal importance to the nasal cavity regarding *Staphylococcus* species colonization, especially *S. aureus* and its multidrug resistance, including in clinically healthy children and adults. These findings highlighted the need for the oral cavity to be included in surveillance and decolonization programs among healthcare professionals and vulnerable patients in order to prevent transmission and bacterial infections. Therefore, this microorganism ability justifies the investigations of the genus *Staphylococcus*, especially *S. aureus*, its antimicrobial resistance patterns, and its relation to rehabilitative interventions and variants in patients with CLP. 

This study aimed to investigate the occurrence of *Staphylococcus* species, especially *S. aureus* and its methicillin resistance profile, in orofacial clefts (pre-surgery) and oral cavities rehabilitated surgically (post-surgery). This study is conducted at different stages of the surgical rehabilitation treatment of patients with CLP, from hospitalization (before asepsis, immediately after asepsis, and immediately after surgical rehabilitation) to their first clinical follow-up after surgery. It also explores the epidemiological correlations between the investigated pathogens and host characteristics as a secondary objective. This study indicated a positive relationship between patients with clefts and the presence of *S. aureus* in their oral microbiota, posing a risk to surgical rehabilitation procedures, which can be minimized with previous asepsis. It was also observed quantitative and qualitative microbial changes according to surgical interventions, corroborating the reduction in the risk of infections and improvement in the quality of life of the patients.

## 2. Materials and Methods

### 2.1. Subjects

**Figure 1:**
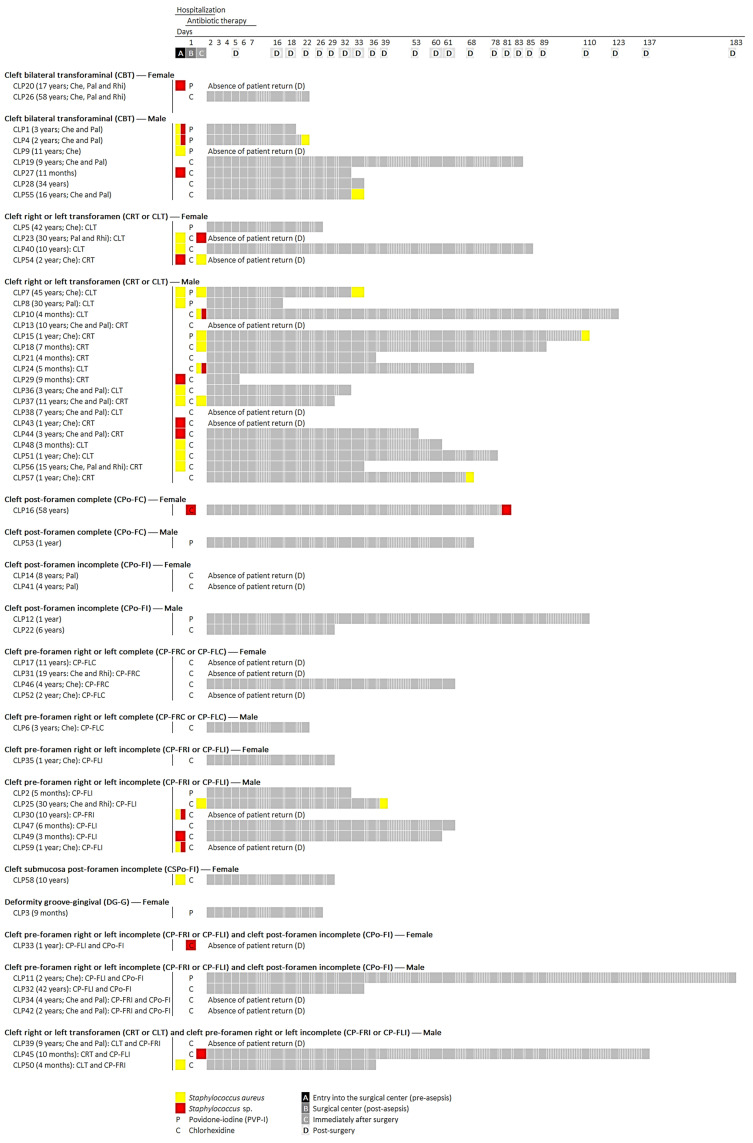
Experimental design and profile of oral colonization by *Staphylococcus* species in patients with orofacial clefts under surgical rehabilitation regarding medical and dental clinical follow-up. Hospitalization period (2 days) and return (≥5 and ≤183 days, mean of 54.2 ± 37.6 days) of patients: (sampling period A) admission to surgical ward prior to asepsis; (sampling period B) prior to surgical procedure and im-mediately after asepsis with PVP-I or chlorhexidine; (sampling period C) immediately after surgical rehabilitation; and (sampling period D) at the first patient return to the *Centro Pró-Sorriso aos Portadores de Fissuras Labial e Palatina*, Alfenas, MG, Brazil. The antibiotic therapy prescribed was administered with first generation cephalosporins (cefazolin or cephalothin) during the first 7 days after hospitalization.

The study involved 59 patients with CLP, with and without surgical history, aged between 3 months and 58 years (mean of 10.1 ± 14.6 years old), both male and female (17 women: mean of 16.3 ± 19.3 years old; 42 men: mean of 7.6 ± 11.6 years old), clinically indicated for surgical rehabilitation. The patients were diagnosed according to the clinical classification of orofacial clefts [1,3] and remained under constant medical and dental clinical follow-up in the Clinics of the School of Dentistry of the José do Rosário Vellano University (UNIFENAS)—Centro Pró-Sorriso aos Portadores de Fissuras Labial e Palatina, of the municipality of Alfenas, state of Minas Gerais (southeast region), Brazil. The patient population was stratified (age group, gender, types of orofacial clefts, surgical history, and types of previous surgical rehabilitation) and statistically analyzed (Figure 1 and Table S1). This research was conducted in accordance with Resolution No. 466/2012 of the National Health Council and approved by the Research Ethics Committee of the Piracicaba Dental School, University of Campinas (FOP/UNICAMP) (Protocol No. 093/2014, CAAE 34875614.0.0000.5418). Informed consent was obtained in written form from all individual participants and/or legal guardians included in the study.

### 2.2. Sampling

Microbiological samples were obtained using the method described previously [25], with some adaptations [14]. For each patient with an orofacial cleft, the samples were collected (pre-surgery: orofacial clefts; post-surgery: oral cavity rehabilitated surgically) using a sterile swab in the presence of a physician and maintained in polypropylene tubes (Corning 50 mL centrifuge tubes, Merck KGaA, Darmstadt, Germany) containing 10 mL of sterile PBS solution (100 mM NaCl, 100 mM NaH2PO4, pH 7.2). Then, these samples were properly transported (4 °C) to the Laboratory of Oral Microbiology and Immunology, School of Dentistry of Piracicaba, State University of Campinas (FOP/UNICAMP). Microbiological samples were obtained at 4 different times between hospitalization (2 days) and return (≥5 and ≤183 days, mean of 54.2 ± 37.6 days) of patients in the period from February 2019 to February 2020: (A) admission to surgical ward prior to asepsis (orofacial cleft sampling); (B) prior to surgical procedure and immediately after asepsis with PVP-I or chlorhexidine (orofacial cleft sampling); (C) immediately after surgical rehabilitation (oral cavity rehabilitated surgically sampling); and (D) at the first patient return to the *Centro Pró-Sorriso aos Portadores de Fissuras Labial e Palatina*, Alfenas, MG, Brazil (oral cavity rehabilitated surgically sampling). The antibiotic therapy prescribed was administered with first generation cephalosporins (cefazolin or cephalothin) during the first 7 days after hospitalization (Figure 1 and Table S1).

### 2.3. Bacterial Isolation

The isolation and preliminary identification of *Staphylococcus* species were based on the characteristics of the bacterial colonies on the MSA selective culture medium (Mannitol Salt Phenol Red Agar, Merck KGaA), Gram staining, catalase test, coagulase test (Coagu-Plasma, Laborclin Produtos para Laboratório), clumping factor A test (Staphy Test, Probac do Brasil Produtos Bacteriológicas), and Voges–Proskauer test [26,27].

The microbiological samples were centrifuged at 1700× *g* for 10 min (Centrifuge, model 5810R, Eppendorf, Darmstadt, Germany). The supernatant was discarded, and the sediments were resuspended in 1 mL of sterile PBS solution (100 mM NaCl, 100 mM NaH_2_PO_4_, pH 7.2), resulting in 10× concentrated sample. Soon after, the sediments were transferred to 2 mL microtubes (Axygen 2.0 mL microcentrifuge tube and cap, Corning, Merck KGaA, Darmstadt, Germany) and shaken in vortex for 30 s [14,25]. Up to 10 mannitol-positive and -negative colonies, indicative of *S. aureus* (presence of yellow halo around colonies) and *Staphylococcus* spp., respectively, were selected at random by sampling, cultivated in BHI culture medium (Brain Heart Infusion Agar, Merck KGaA), and aerobically incubated at 37 °C for 48 h to obtain pure colonies [28]. These isolates were stored (i) in tubes containing 5 mL of BHI agar at −4 °C and (ii) in tubes containing 3 mL of BHI broth supplemented with glycerol (cryopreservation: final concentration of 15%) at –70 °C for short and long periods, respectively. Such procedures enabled access to these cultures in the course of the analyses [29]. The identity of the microbial species was confirmed by MALDI-TOF MS analyses (Matrix-Assisted Laser Desorption Ionization-Time-of-Fight Mass Spectrometry).

### 2.4. MALDI-TOF MS

The identification of the clinical bacterial species was also performed by Matrix-Assisted Laser Desorption Ionization-Time-of-Fight Mass Spectrometry (MALDI-TOF MS), as previously reported [30,31,32].

Initially, clinical bacteria isolates were grown in BHI agar culture medium at 37 °C for 18 h and subjected to their analyte extraction procedures. The cell biomasses were transferred with the help of a disposable loop (10 μL) to 1.5 mL tubes (Eppendorf Safe-Lock Tubes, Eppendorf, Merck KGaA, Darmstadt, Germany) containing 300 μL of type 1 water (Milli-Q Direct 8 System, Millipore Ind. & Comm.) and vortexed for 30 s. Then, aliquots of 900 μL ethanol (Ethyl alcohol pure, Merck KGaA, Darmstadt, Germany) were added to the tubes and again vortexed for 30 s. This mixture was centrifuged at 12,000× *g* for 2 min at –4 °C (Centrifuge, model 5810R, Eppendorf do Brasil Ltd.a., São Paulo, SP, Brazil). Soon after, volumes of 50 μL of 70% formic acid in water type 1 (Formic acid, Merck KGaA, Darmstadt, Germany) were added to the pellets and vortexed for 30 s. Then, aliquots of 50 μL of acetonitrile (Acetonitrile, Merck KGaA, Darmstadt, Germany) were added to the previous mixtures, homogenized using a pipette, and centrifuged at 12,000× *g* for 2 min at −4 °C. The supernatants containing the microbial analytes were transferred to new 1.5 mL Eppendorf tubes and stored at –70 °C until the time of use (<2 months).

For making the 96-spot target plate MSP (Micro SCOUT Plates), 600 nL (0.6 μL) aliquots of the analyte supernatant from each sample were transferred to the surface (spot) of the plate. Then, 1 μL volume of matrix solution [0.01 g of α-Cyano-4-hydroxycinnamic acid (Merck KGaA) in 1 mL of ethanol, acetonitrile, and 10% trifluoroacetic acid in a 1:1:1 ratio—organic solution: 33.3% ethanol (Ethyl alcohol pure, Merck KGaA), 33.3% acetonitrile (Acetonitrile, Merck KGaA), and 33.3% of 10% trifluoroacetic acid (Trifluoroacetic acid, Merck KGaA)], freshly prepared and kept under refrigeration on ice, was added to each nearly dry (spot) surface and mixed thoroughly.

The MSP target plate was kept at room temperature for complete drying and analyzed by MALDI-TOF MS (Maldi-tof Microflex LT spectrometer, Bruker Daltonics GmbH & Co. KG, Bremen, Germany). Sampling of each microbial isolate was analyzed in triplicate (three sequential spots) to ensure reproducibility of data and assay. *Escherichia coli* DH5 alpha was used as test standard (Bruker Daltonics) and for assay calibration. Mass spectra were processed using MALDI Biotyper 3.0 software (Bruker Daltonics) for clinical microbial identification.

### 2.5. Discrimination of MRSA by MALDI-TOF MS

The identification of MRSA (methicillin-resistant *Staphylococcus aureus*) and MSSA (methicillin-sensitive *Staphylococcus aureus*) clinical strains was performed by the Decision Tree method, as previously reported [33]. This method establishes 3 major nodes (Nodes 1, 2, and 3 for MRSA) and 2 terminal nodes (Node 4 for MSSA and Node 5 for *S. aureus* with an undefined profile of methicillin resistance) based on the mass spectra (MALDI-TOF) of 11 peaks (1975, 2194, 2339, 2410, 2592, 2631, 3890, 4607, 5053, 5541, and 6594 *m*/*z*). In summary, Node 1 ^MRSA^ was also used for the classification of SCC*mec* type IV (presence of peak at 5541 *m*/*z* and absence of peak at 5053 *m*/*z*). Node 2 ^MRSA^ was also used for the classification of SCC*mec* type III (concurrent presence of peaks at 2410 and 4607 *m*/*z*). Node 3 was used for the classification of MRSA clinical isolates (presence of one or more peaks at 1975, 2410, 2592, 3890, 4607, or 6594 *m*/*z*—known as R peaks—associated with the absence of peaks at 2194, 2339, and 2631 *m*/*z*—known as S peaks) and also for the classification of SCC*mec* type II based on peaks at 1975, 2592, or 3890 *m*/*z*. Terminal Node 4 was used for classification of MSSA clinical isolates (absence of R peaks). Terminal Node 5 was used to declassify clinical isolates (concomitant presence of one or more R and S peaks) regarding the resistance or sensitivity profile to methicillin.

### 2.6. Data Analysis

The data were subjected to multivariate statistics using SAS software version 9.2, principal component analysis (PCA) and interactive biplot construction, cluster analysis (CA), and dendrogram interpretation (threshold ≤ 0.5) [8].

## 3. Results

Patients with orofacial clefts were evaluated for oral staphylococcal colonization throughout their surgical rehabilitation: period of admission to the operating room (before asepsis) (period A), immediately after asepsis (period B), immediately after surgical rehabilitation (period C), and post-surgery follow-up (period D: ≥5 and ≤183 days, mean of 54.2 ± 37.6 days). Microbiological methods served to isolate and presumptively identify *Staphylococcus* species (MSA selective culture medium, Gram stain, catalase, coagulase, clumping factor A, and Voges–Proskauer tests), whereas the MALDI-TOF MS technology performed the species-specific characterization. Overall, 33 (55.9%) of the 59 patients showed oral staphylococcal colonization in:

One sampling period (25 ^42.4%^ patients): Period A ^30.5%^ (CLP1, CLP8, CLP9, CLP20, CLP27, CLP29, CLP30, CLP36, CLP40, CLP43, CLP44, CLP48, CLP49, CLP50, CLP51, CLP56, CLP58, and CLP59), Period B ^1.7%^ (CLP33), Period C ^6.8%^ (CLP10, CLP18, CLP24, and CLP45), and Period D ^3.4%^ (CLP55 and CLP57);

Two sampling periods (7 ^11.9%^ patients): Periods AC ^5.1%^ (CLP23, CLP37, and CLP54), Periods AD ^1.7%^ (CLP4), Periods BC ^1.7%^ (CLP16), and Periods CD ^3.4%^ (CLP15 and CLP25); and

Three sampling periods (1 ^1.7%^ patient): Periods ACD ^1.7%^ (CLP7).

Of the 169 identified clinical isolates, *S. aureus* (*n* = 122 ^72.2%^) was highly prevalent in the population of patients with orofacial clefts over the investigated periods, followed by *S. saprophyticus* (*n* = 19 ^11.2%^), *S. epidermidis* (*n* = 17 ^10.1%^), *S. sciuri* (*n* = 5 ^3.0%^), *S. haemolyticus* (*n* = 2 ^1.2%^) and *S. lentus* (*n* = 2 ^1.2%^), *S. arlettae* (*n* = 1 ^0.6%^), and *S. warneri* (*n* = 1 ^0.6%^) (Tables S1 and S2, and Figure 1).

### 3.1. Subjects and Staphylococcus Species

**Figure 2:**
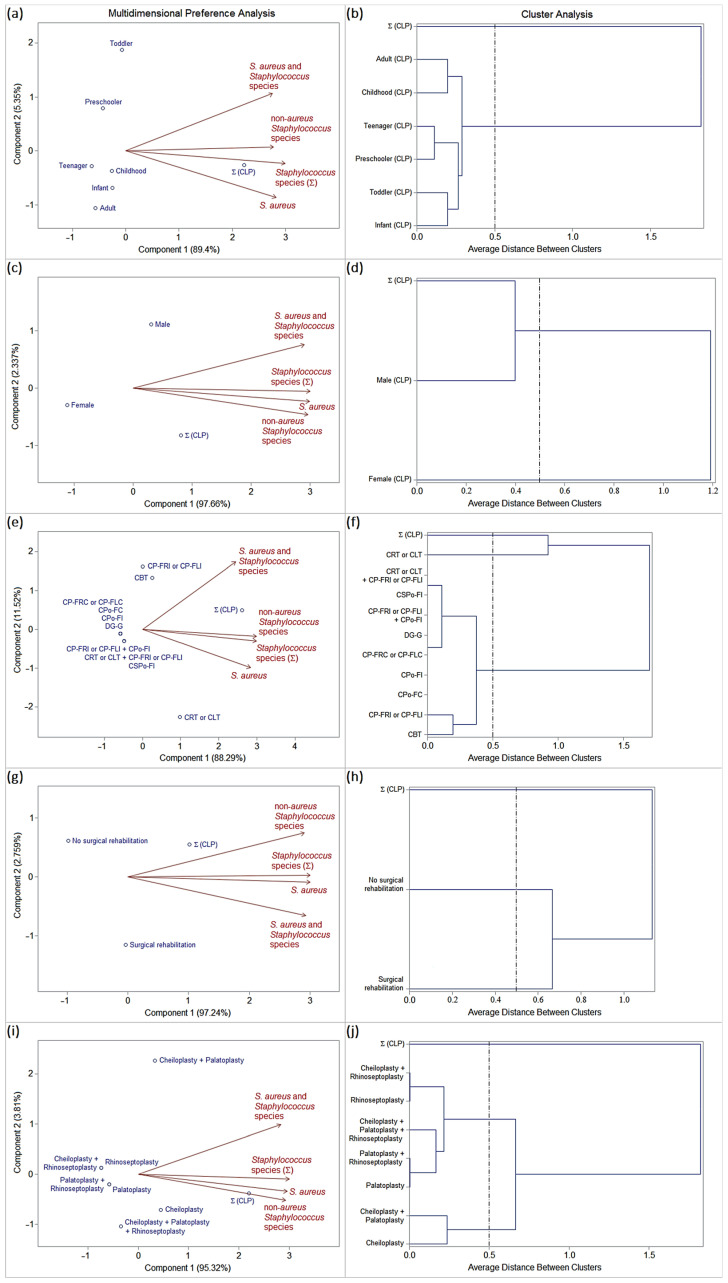
Biplots (PCA) and dendrograms (CA) analysis (SAS^®^ version 9.2; threshold ≤ 0.5) based on the frequency *Staphylococcus* species isolated from patients with orofacial clefts in period A. Epidemiological variables: (**a**,**b**) age group, (**c**,**d**) gender, (**e**,**f**) types of orofacial clefts, (**g**,**h**) surgical history, and (**i**,**j**) types of previous surgical rehabilitation.

Considering the period of admission to the operating room (period A: before asepsis), 23 (39%) patients with orofacial clefts showed staphylococcal oral colonization, twelve (20.3%) patients being colonized exclusively by *S. aureus* (homogeneous colonization: CLP7, CLP8, CLP9, CLP23, CLP36, CLP37, CLP40, CLP48, CLP50, CLP51, CLP56, and CLP58), four (6.8%) colonized by *S. aureus* and *Staphylococcus* spp. (heterogeneous colonization: CLP1, CLP4, CLP30, and CLP59), and seven (11.9%) colonized by *S.* non-*aureus* (homogeneous or heterogeneous colonization: CLP20, CLP27, CLP29, CLP43, CLP44, CLP49, and CLP54) (Tables S1 and S2, and Table 1). In addition, patients admitted to the operating room (period A) started antibiotic therapy according to medical prescription (first-generation cephalosporins for 7 days from hospitalization: cefazolin or cephalothin). Data on the frequency of oral staphylococcal species (mono- and multi-colonization) in patients with orofacial clefts and their epidemiological variables (age group, gender, types of orofacial clefts, surgical history, and types of previous surgical rehabilitation) were subjected to multivariate statistics using SAS^®^ version 9.2 [principal component analysis (PCA) and interactive biplot construction; cluster analysis (CA) and dendrogram interpretation (threshold ≤ 0.5)], which showed similar profiles, as follows (Table 1 and Figure 2a–j).

Age group (Figure 2a,b):

The frequencies of oral colonization by *S. aureus* (homogeneous colonization), *S. aureus* and *Staphylococcus* spp. (heterogeneous colonization), and *S.* non-*aureus* (homogeneous or heterogeneous colonization) were considered uniform among the age groups stratified into infants, toddlers, preschoolers, childhoods, teenagers, and adults.

Gender (Figure 2c,d):

The male gender was characterized by a high frequency of oral colonization by *S. aureus* (homogeneous colonization), *S. aureus* and *Staphylococcus* spp. (heterogeneous colonization), and *S.* non-*aureus* (homogeneous or heterogeneous colonization).

The female gender was characterized by a low frequency of oral staphylococcal colonization.

Types of orofacial clefts (Figure 2e,f):

CRT or CLT was characterized by a high frequency of oral staphylococcal colonization, especially by *S. aureus* (mono-colonization or homogeneous colonization), followed by *S.* non-*aureus* (homogeneous or heterogeneous colonization).

The other types of orofacial clefts were characterized by a low (CBT, CP-FRI or CP-FLI, CSPo-FI, and CRT or CLT + CP-FRI or CP-FLI) or null (CPo-FC, CPo-FI, CP-FRC or CP-FLC, DG-G, and CP-FRI or CP-FLI + CPo-FI) frequency of staphylococcal oral colonization.

Surgical history (Figure 2g,h):

Patients with a history of surgical rehabilitation were characterized by a high frequency of oral colonization by *S. aureus* (homogeneous colonization), *S. aureus* and *Staphylococcus* spp. (heterogeneous colonization), and *S.* non-*aureus* (homogeneous or heterogeneous colonization).

Patients with no history of surgical rehabilitation were characterized by a low frequency of oral staphylococcal colonization.

Types of previous surgical rehabilitation (Figure 2i,j):

Patients who had previously undergone only cheiloplasty or cheiloplasty + palatoplasty procedures were characterized by a high frequency of oral colonization by *S. aureus* (homogeneous colonization), *S. aureus* and *Staphylococcus* spp. (heterogeneous colonization), and *S.* non-*aureus* (homogeneous or heterogeneous colonization).

Patients who had previously undergone other types of surgical procedures were characterized by a low frequency of oral staphylococcal colonization.

**Figure 3:**
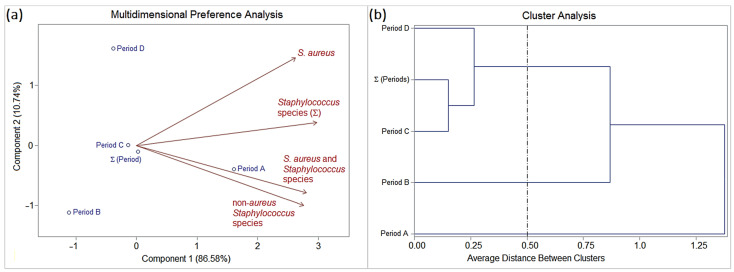
(**a**) Biplot (PCA) and (**b**) dendrogram (CA) analysis (SAS^®^ version 9.2; threshold ≤ 0.5) based on the frequency *Staphylococcus* species (mono- and multi-colonization; homogeneous and heterogeneous colonization) isolated from patients with orofacial clefts. Epidemiological variable: “periods of hospitalization” and “surgical asepsis procedures”.

Qualitative and quantitative changes in the profiles of oral staphylococcal colonization were observed between sampling periods A (before surgical asepsis), B (immediately after surgical asepsis with chlorhexidine or PVPI), C (immediately after surgical rehabilitation), and D (first medical return of the patient after surgery: 5–183 days, mean 54.2 ± 37.6 days). Overall, of the twenty-three (39%) patients positive for oral *Staphylococcus* species in period A, only two (3.4%) patients tested positive in period B, whereas periods C and D recorded an increase of ten (16.9%) and seven (17.1%) patients colonized by *Staphylococcus* spp., respectively. The two patients in period B were colonized exclusively by *S.* non-*aureus* (homogeneous colonization by *S. saprophyticus*: patients CLP16 and CLP33). Period C recorded six (10.2%) patients exclusively colonized by *S. aureus* (homogeneous colonization: patients CLP7, CLP15, CLP18, CLP25, CLP37, and CLP54), two (3.4%) colonized by *S. aureus* and *Staphylococcus* spp. (other staphylococcal species) (heterogeneous colonization: patients CLP10 and CLP24), and two (3.4%) colonized by *S.* non-*aureus* (homogeneous colonization: patients CLP23 and CLP45). Period D recorded six (14.6%) patients exclusively colonized by *S. aureus* (homogeneous colonization: patients CLP4, CLP7, CLP15, CLP25, CLP55, and CLP57) and only 1 (2.4%) colonized by *S.* non-*aureus* (homogeneous colonization: patient CLP16). Surgical asepsis with chlorhexidine and PVPI occurred in 46 and 13 patients, respectively. However, the two groups of patients stratified for surgical asepsis procedures (chlorhexidine or PVPI) showed equivalent profiles in the frequencies of oral staphylococcal colonization in all periods (Tables S1 and S2, and Table 2). Data on the frequency of oral staphylococcal species (mono- and multi-colonization; homogeneous and heterogeneous colonization) in patients with orofacial clefts and their epidemiological variables (hospitalization periods and surgical asepsis procedures) were subjected to multivariate statistics using SAS^®^ version 9.2 [principal component analysis (PCA) and interactive biplot construction; cluster analysis (CA) and dendrogram interpretation (threshold ≤ 0.5)], which showed similarity profiles as follows (Table 2 and Figure 3):

Period A (before surgical asepsis) was characterized by the highest frequency of oral colonization by *S. aureus* (homogeneous colonization), followed by *S.* non-*aureus* (homogeneous or heterogeneous colonization), and *S. aureus* + *Staphylococcus* spp. (heterogeneous colonization).

Period B was characterized by zero or low frequency of oral staphylococcal colonization, in particular homogeneous colonization by *S.* non-*aureus*.

Periods C and D were characterized by frequencies intermediate to periods A and B of oral staphylococcal colonization, especially by *S. aureus*, followed by *S. aureus* + *Staphylococcus* spp. and *S.* non-*aureus*.

The interpretation of quantitative and qualitative data showed the dynamics of oral staphylococcal colonization in patients with orofacial clefts throughout the periods of hospitalization and medical follow-up. Period A (baseline) began with 36 (61%) cases of aseptic maintenance and 23 (39%) cases of septic maintenance, corresponding to the frequencies of absence and presence of oral staphylococcal colonization in the patients, respectively (Tables S1 and S2, and Table 3). The following are compared with period A:

Period B had thirty-four (57.6%) cases of aseptic maintenance, twenty-three (39%) cases of septic elimination, and two (3.4%) cases of septic neocolonization;

Period C showed thirty (50.8%) cases of aseptic maintenance, nineteen (32.2%) cases of septic elimination, six (10.2%) cases of septic neocolonization, two (3.4%) cases of septic maintenance, and two (3.4%) cases of septic elimination and septic neocolonization simultaneously;

Period D showed twenty (48.8%) cases of aseptic maintenance, fourteen (34.1%) cases of septic elimination, five (12.2%) cases of septic neocolonization, one (2.4%) case of septic maintenance, and one (2.4%) case of septic elimination and septic maintenance simultaneously, totaling forty-one (69.5%) comparative cases in this period. Therefore, 18 (30.5%) cases eluded comparison due to the lack of patient return up to the maximum period of the investigation (183 days).

**Figure 4:**
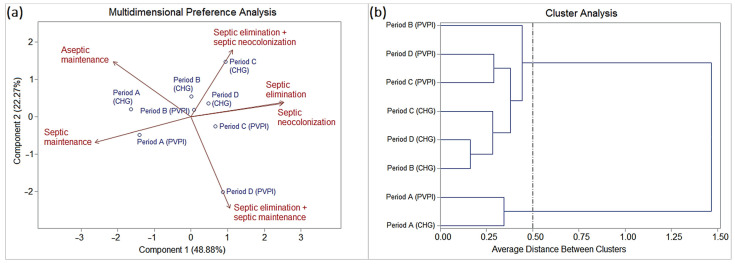
(**a**) Biplot (PCA) and (**b**) dendrogram (CA) analysis (SAS^®^ version 9.2; threshold ≤ 0.5) based on the frequency of the dynamics of oral colonization by *Staphylococcus* species isolated from patients with orofacial clefts throughout the pre- and post-surgical periods (periods A, B, C, and D).

Data on the frequency of oral staphylococcal species (mono- and multi-colonization) in patients with orofacial clefts and their epidemiological variables (dynamics between periods and surgical asepsis procedures) were subjected to multivariate statistics using SAS^®^ version 9.2 [principal component analysis (PCA) and interactive biplot construction; cluster analysis (CA) and dendrogram interpretation (threshold ≤ 0.5)], which showed similar profiles as follows (Table 3 and Figure 4):

The chlorhexidine and PVPI groups showed no statistically significant differences.

Period A was characterized by a higher frequency of onset (baseline) in the cases of septic maintenance (SM) and aseptic maintenance (AM) than the other periods.

Periods B, C, and D were characterized by (i) decreasing frequencies in cases of aseptic maintenance (AM), especially in periods C and D; (ii) high frequencies in cases of septic elimination (SE), especially in period B, followed by C and D; (iii) a slight increase in the frequencies of cases of septic neocolonization (SN), especially in periods C and D, followed by B; (iv) low frequencies (periods C and D) or null frequencies (period B) in cases of septic maintenance (SM); and a slight frequency for simultaneous cases of SE and SN (period C) and SE and SM (period D).

### 3.2. MRSA Diagnosed by MALDI-TOF MS

**Figure 5:**
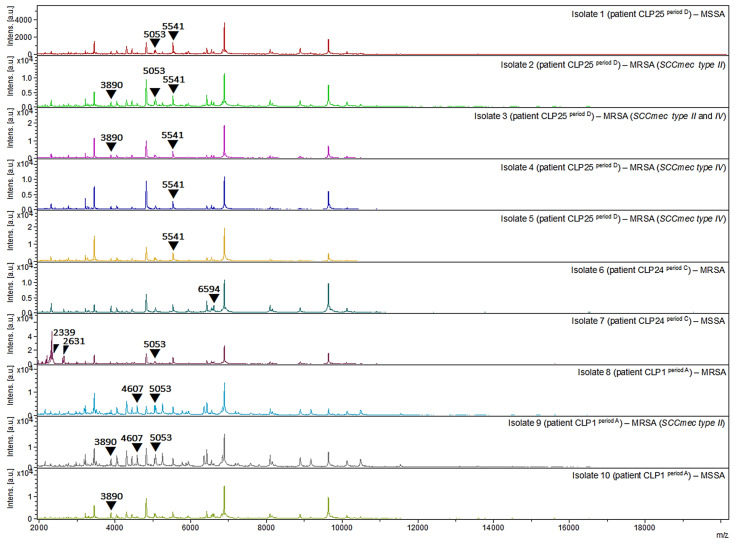
Protein spectra profiles (2000–20,000 *m*/*z*) of MRSA and MSSA clinical strains isolated from oral cavity of three patients with orofacial clefts. CLP1: MRSA (isolates 8 ^period A^ and 9 ^period A^) and MSSA (isolate 10 ^period A^); CLP24: MRSA (isolate 6 ^period C^) and MSSA (isolate 7 ^period C^); CLP25: MRSA (isolates 2 ^period D^, 3 ^period D^, 4 ^period D^, and 5 ^period D^) and MSSA (isolate 1 ^period D^).

Of the 122 clinical species of *S. aureus*, from the oral cavity of 24 hospitalized patients with orofacial clefts (CLP1, CLP4, CLP7, CLP8, CLP9, CLP10, CLP15, CLP18, CLP23, CLP24, CLP25, CLP30, CLP36, CLP37, CLP40, CLP48, CLP50, CLP51, CLP54, CLP55, CLP56, CLP57, CLP58, and CLP59) and analyzed by the Decision Tree method (i.e., based on the mass spectra of 11 peaks: 1975, 2194, 2339, 2410, 2592, 2631, 3890, 4607, 5053, 5541, and 6594 *m*/*z*), 74 (60.7%), 25 (20.5%), and 23 (18.9%) clinical isolates were identified as MRSA (21 patients), MSSA (21 patients), and unclassified (3 patients), respectively (Table 4 and Figure 5).

Among the MRSA clinical isolates, 32 (26.2%) were identified at Node 1 and classified as SCC*mec* type IV (presence of the peak at 5541 *m*/*z* and absence of the peak at 5053 *m*/*z*), and 51 (41.8%) were identified at Node 3 (presence of one or more R-peaks: 1975, 2410, 2592, 3890, 4607, or 6594 *m*/*z*; associated with the absence of the S-peaks: 2194, 2339, and 2631 *m*/*z*), 36 (29.5%) of which were classified as SCC*mec* type II (presence of peaks at 1975, 2592, or 3890 *m*/*z*). No clinical isolates were identified in Node 2 and classified as SCC*mec* type III (concomitant presence of peaks at 2410 and 4607 *m*/*z*). Nine (7.4%) clinical isolates showed two classifications and were concomitantly identified as SCC*mec* type II and IV: isolates 3 (CLP25, period D, 2.347 ^BCR^), 34 (CLP9, period A, 2.206 ^BCR^), 35 (CLP9, period A, 2.178 ^BCR^), 41 (CLP37, period A, 2.015 ^BCR^), 51 (CLP25, period C, 2.034 ^BCR^), 53 (CLP23, period A, 2.137 ^BCR^), 54 (CLP23, period A, 2.140 ^BCR^), 55 (CLP23, period A, 2.145 ^BCR^), and 56 (CLP23, period A, 2.356 ^BCR^).

The frequency of MRSA clinical isolates among patients with orofacial clefts ranged from 20 to 100%: CLP40 (*n* = 1 ^20%^), CLP56 (*n* = 1 ^20%^), CLP7 (*n* = 3 ^25%^), CLP51 (*n* = 2 ^40%^), CLP54 (*n* = 2 ^40%^), CLP15 (*n* = 4 ^44.4%^), CLP24 (*n* = 1 ^50%^), CLP59 (*n* = 1 ^50%^), CLP25 (*n* = 9 ^60%^), CLP48 (*n* = 3 ^60%^), CLP36 (*n* = 2 ^66.7%^), CLP4 (*n* = 6 ^66.7%^), CLP8 (*n* = 4 ^80%^), CLP23 (*n* = 4 ^80%^), CLP37 (*n* = 8 ^88.9%^), CLP1 (*n* = 9 ^100%^), CLP9 (*n* = 5 ^100%^), CLP50 (*n* = 1 ^100%^), CLP55 (*n* = 2 ^100%^), CLP57 (*n* = 5 ^100%^), and CLP58 (*n* = 1 ^100%^). In three patients, clinical isolates of *S. aureus* were considered unclassified (CLP10, CLP18, and CLP30).

Period A showed a higher frequency of MRSA clinical isolates (*n* = 47 ^38.5%^), with 16 (13.1%) isolates classified as SCC*mec* type IV and 20 (16.4%) as SCC*mec* type II. Periods C and D showed identical frequencies of MRSA clinical isolates (*n* = 18 ^14.8%^), with seven (5.7%) isolates classified as SCC*mec* type IV and nine (7.4%) as SCC*mec* type II in period C, and nine (7.4%) isolates classified as SCC*mec* type IV and seven (5.7%) as SCC*mec* type II in period D. Among the MSSA clinical species, fourteen (11.5%), eight (6.6%), and three (2.5%) isolates were observed in periods A, C, and D, respectively. Among the unclassified isolates, eleven (9.0%), eight (6.6%), and four (3.3%) isolates were observed in periods A, C, and D, respectively.

## 4. Discussion

### 4.1. Subjects and Staphylococcus Species

A total of 59 patients with orofacial clefts were analyzed for oral staphylococcal colonization during four stages of surgical rehabilitation, from hospitalization to their first return to the medical clinic: period of admission to the operating room and prior to asepsis (period A), immediately after asepsis (period B), immediately after surgical rehabilitation (period C), and patient return after surgery (period D: 5–183 days). Of these, 33 (55.9%) patients showed oral staphylococcal colonization in one, two, or three sampling stages, totaling 169 clinical *Staphylococcus* species identified. High prevalence was reported for *S. aureus* (72.2%), followed by *S. saprophyticus* (11.2%), *S. epidermidis* (10.1%), *S. sciuri* (3.0%), *S. haemolyticus* (1.2%), *S. lentus* (1.2%), *S. arlettae* (0.6%), and *S. warneri* (0.6%).

The anatomical structures and functions of the oral and nasal cavities are affected by orofacial clefts, in addition to comprising important microbial entry ports to the human organism [34]. The anatomical rearrangement can greatly affect the characteristics of the resident microbiota, altering the microenvironment, and, therefore, changes related to orofacial clefts and their anatomical niches can influence oral and nasal bacteria qualitatively and quantitatively [35]. Microbiological culture and molecular methods of genomic sequencing used in epidemiological studies have shown an increased proportion of opportunistic pathogens in the oral cavity of patients with orofacial clefts, such as: *Acinetobacter*, *Candida*, *Citrobacter*, *Enterobacter*, *Enterococcus*, *Escherichia*, *Gemella*, *Klebsiella*, *Lactobacillus*, *Lactococcus*, *Moraxella*, *Neisseria*, *Serratia,* and *Staphylococcus*, including the high incidence of *S. aureus* and *Streptococcus* [8,9,10,11,12,13,14,15]. Furthermore, these clefts correspond to a condition of anomaly that can lead to loss of continuity in the labial, alveolar, and palatine tissues of the maxilla, which indicates the need for surgical procedures for functional and aesthetic rehabilitation of the patient. Therefore, these patients present dysregulation of the balance of the oral microbiota, with pathological migration of bacteria between the oral and nasal cavities, which corresponds to a population with high rates of microorganisms in these regions [13], as proven in this study and other recent investigations [8,14].

In the first stage of hospitalization, prior to surgical asepsis (period A), oral staphylococcal colonization was observed in 23 (39%) patients with orofacial clefts. Of these, most patients (20.3%) were colonized exclusively by *S. aureus* (homogeneous colonization), followed by a minority of patients colonized by *S. aureus* and other species of the *Staphylococcus* genus (heterogeneous colonization) (6.8%) or by *S.* non-*aureus* (homogeneous or heterogeneous colonization) (11.9%). Moreover, the frequency of oral *Staphylococcus* species was similar to that of oral opportunistic *Candida* species (i.e., 39.1%; potentially virulent biotypes, especially for SAPs, and showing resistance to fluconazole and sensitivity or clinical compatibility to the polyenes amphotericin and nystatin) from groups of infants and children with orofacial clefts admitted to the hospital under antibiotic prophylaxis with cephalosporins of first generation (cefazolin or cephalothin) and prior to surgical asepsis [14]. It is important to note that all patients admitted to the operating room also started preventive antibiotic therapy with first-generation cephalosporins (cefazolin or cephalothin). Correlation analyses of the data showed that the profiles of oral staphylococcal colonization (homogeneous and/or heterogeneous; mono-colonization and/or multi-colonization) are (i) uniform among age groups, (ii) highly more frequent in men than in women, (iii) highly frequent in patients with CRT or CLT, especially by *S. aureus* in its homogeneous configuration (mono-colonization), as opposed to other types of orofacial clefts with low or no frequencies, and (iv) highly frequent in patients with a history of surgical rehabilitation, especially in those who have undergone cheiloplasty or cheiloplasty + palatoplasty.

Information on preoperative bacteriology in children with orofacial clefts is found in the literature [36], with special involvement of *S. aureus*. In 158 children diagnosed with labiopalatine syndromic conditions, 101 (64%) were positive for pathogenic bacteria prior to surgery, and 65 (41%) and 30 (19%) were carriers of *S. aureus* and *Streptococcus* β-hemolytics, respectively. Hospital bacterial infections that result from various types of surgeries often result in complications for the clinical health of patients, especially those involving primary cleft lip and palate, with high risks of surgical dehiscence and slow healing processes, in addition to other complications that lead patients to death. Nevertheless, there are several ways to assess the risks of bacterial infections in the post-operative period of these syndromic conditions: physical and clinical examinations of infections, laboratory data, criteria of the Systemic Inflammatory Response Syndrome (SIRS), blood cultures prior to antibiotic therapy, assessments of appropriate antimicrobial therapies to be prescribed, reassessment of antimicrobial therapy daily for relief when appropriate, among others [37]. Unfortunately, the analysis of the risks of infections does not include routine microbiological tests at pre-operative times, in part due to prophylactic antibiotic prescription as a presumed action in reducing the risks of infections in all cases of surgical rehabilitation [38]. Recent guidelines emphasize the importance of microbiological clinical diagnosis, based on the precise identification of pathogens causing infections and on the characterization of their virulence profiles (e.g., antimicrobial resistance detected by susceptibility patterns), which allows the implementation of adequate antibiotic therapy [39,40,41]. The widespread use of broad-spectrum antibiotics without this primary identification probably favored the development of multidrug-resistant strains [42,43]. The significantly reduced risks of post-operative infections by the use of antibiotic prophylaxis have great clinical relevance, but there is still disagreement about the duration of antibiotic therapy and the choice of antibiotics due to failures in the clinical microbiological diagnosis process [44,45].

The drugs prescribed in the course of this study, cefazolin and cephalothin (first-generation cephalosporins), are β-lactams antimicrobial agents with a wide spectrum of action, acting against gram-positive bacteria (*Staphylococcus* spp. and *Streptococcus* spp.) and gram-negative bacteria (*Proteus mirabilis*, *Escherichia coli*, and *Klebsiella pneumoniae*) [46,47]. Cefazolin stands out among antimicrobial drugs since it can be administered less frequently due to its longer half-life (time required for the maximum serum concentration achieved with the administration of a standard dose to be halved) [46]. Additionally, its prescription and clinical performance have been highlighted for years as the drug of choice in perioperative prophylaxis (pre-operative, intra-operative, and post-operative periods) [48]. The single dose of cefazolin immediately before surgery constitutes a prophylactic measure widely prescribed in several surgical procedures [49] and is associated with a reduced risk of post-operative infections and severe clinical complications for patients [50]. On the other hand, a study related to the administration of cephalothin indicates a higher clearance and a shorter half-life than cefazolin and, consequently, faster metabolic elimination [51]. Moreover, the clinical practice guidelines for antimicrobial prophylaxis in surgery report that cefazolin is the drug of choice for surgical prophylaxis in various procedures due to its favorable safety profile and low cost compared with cephalothin [52].

Nasal, sublingual, and oropharyngeal anatomical surfaces in patients with orofacial clefts, prior to and shortly after reconstructive surgeries, also revealed diversity of genera and microbial species, as well as significantly reduced counts of species *Klebsiella*, *Enterobacter*, and *S. aureus* after complete closure of the cleft palate [53,54]. These data corroborate in part with the findings of this study, especially in staphylococcal oral colonization in the post-operative period, strongly indicating the need to adopt effective measures in microbial control and consequent prevention of infections throughout the repair and surgical healing processes and clinical recovery of patients.

The uniformity of the oral staphylococcal colonization profile (homogeneous and/or heterogeneous; mono-colonization and/or multi-colonization) across age groups can be partially observed, according to some literature findings. Higher microbial incidence has been reported in infants, justified by maternal skin contact via breastfeeding as an important factor in the increase of gram-positive and gram-negative bacteria, especially for *Staphylococcus* species, including MRSA [53]. In part, these differences can be explained by the intrinsic characteristics of the sample of patients in each study. The oral microbiota has been characterized by dynamic changes throughout the life of clinically healthy and normal individuals, and some factors may affect the colonization process of microorganisms, such as (i) the presence of biofilms on the surface of soft and hard tissues, (ii) the frequency of microbial exposure (via food, personal contact, animals, and other contaminated inanimate surfaces, among others), (iii) long-term hospital care, and (iv) antibiotic treatment [55]. Thus, changes in the microbial ecological landscape influence the structures and functions of this microbiota [56].

The high frequency of staphylococcal oral colonization in men also corroborates the data on the higher prevalence of oral microbial colonization in them. Studies have documented the immunosuppressive role of testosterone and the increased male propensity for bacterial infections [57,58]. Patients with CRT or CLT also showed high frequencies of oral staphylococcal colonization, especially homogeneous configuration (mono-colonization) by *S. aureus*, followed by homogeneous or heterogeneous colonization (multi-colonization) by *S.* non-*aureus*, unlike the other types of orofacial clefts. These findings correspond to other reports in the literature [53,59] and show the presence of *S. aureus* before surgical rehabilitation events, especially in pre-asepsis, indicating once again the importance of adopting effective measures in pre- and postoperative microbial control.

Similarly, patients with a history of surgical rehabilitation, especially cheiloplasty or cheiloplasty + palatoplasty, showed a high frequency of oral staphylococci in the three colonization profiles (*S. aureus*; *S. aureus* and *Staphylococcus* spp.; and *S.* non-*aureus*). Closure of abnormal oral fissures by surgical repair can restore the normal structure and function of the oronasopharynx [54]. However, the recurrence of surgical procedures due to impairments that are difficult to determine on a case-by-case basis (e.g., surgical dehiscence or intrinsic healing capacity) after primary medical interventions, including surgical asepsis and pre- and postoperative antibiotic prophylaxis, may contribute to the high oral microbial frequency [60,61]. The low microbial frequency and its metabolic activities in surgical anatomical sites can be achieved by efficient microbiological control actions using the association of different compounds (e.g., asepsis with chlorhexidine solution, PVPI) and antibiotic prophylaxis [62]. Moreover, other factors should be considered when choosing the asepsis procedure and treatment to minimize the risks of microbial colonization and oral infections: type and duration of surgery, intra- or extraoral access, possibility of hematoma or edema formation, tissue trauma, and allergies to composites [63]. In addition to the importance of microbial control, it is noteworthy that the bacteremia phenomenon was considered a common event after surgical repair of cleft lip and palate, especially involving potentially pathogenic microorganisms, such as the β-hemolytic streptococcal species of group A and *S. aureus*. These pathogens can cause local infections, complications associated with wound healing, and septicemic shock [64].

Staphylococcal oral colonization profiles (*S. aureus*; *S. aureus* and *Staphylococcus* spp.; and *S.* non-*aureus*) showed changes in frequencies and species-specifics between sampling periods. Of the 23 (39%) patients who were positive for the microbiological diagnosis at the time of hospitalization or surgical pre-asepsis (period A or comparative baseline: higher frequency), only two (3.4%), 10 (16.9%), and seven (17.1%) patients were positive for the microbiological diagnosis in the sequence of medical procedures: (i) immediately after surgical asepsis with chlorhexidine or PVPI (period B: lowest frequency), (ii) immediately postoperative (period C: intermediate frequency), and (iii) the first clinical medical follow-up (period D: intermediate frequency), respectively. As previously mentioned, the antibiotic therapy prescribed to the patients with CLP was administered with first-generation cephalosporins (cefazolin or cephalothin) within 7 days of hospitalization. However, this clinical prophylactic management potentially also interferes with the metabolic expression of resident and transient microbiota, opportunistic and pathogenic, and immunomodulation, resulting in the partial or total effectiveness of prophylaxis, antibiotic therapy, and the prevention of infections. In pathogen selection, for example, the frequent employment of these antimicrobials in hospitalized and preoperative patients tends to modify their intestinal, oropharyngeal, and skin normal microbiotas, favoring colonization and infection by cephalosporin-resistant microorganisms, including *Acinetobacter*, *Enterococcus*, *Pseudomonas,* and *Staphylococcus* species [65]. In microbial expression, clinical isolates of *S. aureus* producing A and C variants of staphylococcal β-lactamase were also associated with deep sternal wound infections after perioperative prophylaxis with cefazolin and cefamandole, respectively. Such isolates were able to hydrolyze the respective cephalosporins rapidly, suggesting that staphylococcal survival may be mediated by the in vivo degradation of the prophylactically administered cephalosporin [66]. In immune modulation, the bactericidal action of β-lactam antibiotics is impaired by phagocytic cells, as the clearance of *S. aureus* from infection-accessible sites to phagocytes was delayed compared with phagocyte-inaccessible sites when treated with cephalothin, emphasizing the challenges in eradicating staphylococcal colonization even with antibiotic therapy [67]. Moreover, surgical asepsis with chlorhexidine and PVPI showed equivalent oral staphylococcal frequencies throughout the succession of those medical-hospital procedures, especially after surgical asepsis. Other studies [68,69,70,71] have also reported low frequencies or elimination of microbial composition in surgical aseptic prophylaxis with these chemical compounds. Chlorhexidine has been used as an antiseptic of epithelial tissue for skin (wounds) and mucous membranes (oral cavity), and its bacteriostatic (low concentration) and bactericidal (high concentration) effects depend on concentrations [68]. This antiseptic acts on microbial plasma membranes and protein precipitation, rupturing microbial cells and causing death due to its mechanism of action, especially in bacteria (gram-positive and gram-negative, aerobic and facultative anaerobic), viruses, and some filamentous and yeast-like fungi [68]. Chemically, chlorhexidine has great solubility potential, an almost immediate action (~15 s), and low irritability [69]. On the other hand, the antiseptic based on 10% polyvinylpyrrolidone plus 1% iodine (PVPI) has stability and adhesion in contact areas and can penetrate via the microbial plasma membrane and oxidation of cellular components, followed by cell death as its action mechanism. Its antiseptic effects occur in just two minutes, but with reduced effects in the presence of alkaline substances and organic matter [70,71]. Thus, the data reported in this study suggest that antibiotic therapy, asepsis procedures, and oral surgical techniques favor the reduction of the frequency of oral staphylococcal colonization in patients with rehabilitated orofacial clefts (post-surgery), as well as influence the composition of bacterial species of the genus *Staphylococcus*.

Notably, the dynamics of oral staphylococcal colonization in patients with orofacial clefts are shown throughout the periods of hospitalization and medical follow-up. The admission of patients to hospitalization (period A, comparative baseline) was reported, with cases of aseptic maintenance (61%) and septic maintenance (39%). Immediate post-asepsis (period B) was reported, with cases of aseptic maintenance (57.6%), septic elimination (39%), and septic neocolonization (3.4%). The immediate postoperative period (period C) was reported with cases of aseptic maintenance (50.8%), septic elimination (32.2%), septic neocolonization (10.2%), septic maintenance (3.4%), and simultaneous septic elimination and septic neocolonization (3.4%). Finally, the first follow-up visit and clinical follow-up of patients (period D) was reported with cases of aseptic maintenance (48.8%), septic elimination (34.1%), septic neocolonization (12.2%), septic maintenance (2.4%), and simultaneous septic elimination and septic maintenance (2.4%). Moreover, the clinical study period (≤183 days) showed (30.5%) patients’ lack of first follow-up (period D). This phenomenon can be explained at least in part by the intrinsic characteristics of the population assisted at the Centro Pró-Sorriso aos Portadores de Fissuras Labial e Palatina (e.g., living in distant geographic locations, travel and transportation difficulties, socioeconomic conditions, among others). The set of these observations again suggests that the antiseptics chlorhexidine and PVPI used in asepsis procedures also contribute to the significant reduction of oral staphylococcal colonization in patients with orofacial clefts undergoing surgical rehabilitation (i.e., they contribute to this staphylococcal dynamic, especially after immediate asepsis). Changes in the oral staphylococcal microbiota in patients with orofacial clefts also begin after surgical rehabilitation and show a colonization dynamic throughout treatment and medical follow-up, which is influenced by the following:

(i) Imbalance in aseptic maintenance, which generally occurs due to the phenomena of oral decontamination (septic elimination), colonization of the mucosa previously uncontaminated by the genus Staphylococcus after surgical anatomical correction (septic neocolonization), persistence of the genus Staphylococcus in the oral mucosa after surgical treatment (septic maintenance), or even the simultaneous presence of these phenomena (SE and SN simultaneously; SE and SM simultaneously);

(ii) Efficiency of surgical asepsis (septic elimination) on the oral staphylococcal microbiota in all cases of Staphylococcus-positive patients, but possible rare cases of staphylococcal oral contamination at the time of asepsis (septic neocolonization) are likely to occur in Staphylococcus-negative patients;

(iii) Staphylococcal neocolonization that, although less often, appears in new anatomical habitats surgically rehabilitated oral cavity in some patients previously Staphylococcus-negative (septic neocolonization) or *Staphylococcus*-positive (SE and SN simultaneously), after surgical treatment and/or in the course of medical follow-up;

(iv) Total (septic maintenance) or partial maintenance (SE and MS simultaneously) of certain oral staphylococcal species that are detected in some surgically rehabilitated patients either soon after surgical treatment and/or in the course of medical follow-up.

Thus, the elimination or significant reduction of potentially pathogenic microorganisms in human hosts should be aimed at by effective surgical and hospital asepsis actions (i.e., technically adequate procedures for surgical rehabilitation) to minimally ensure the risks of various contaminations, cross-transmissions, and infectious processes and thus favor the reestablishment of anatomical structures and their functions and patients’ health and well-being [7]. Thus, these research results add important concepts for the actions and objectives mentioned above, especially for the microbiota belonging to the genus *Staphylococcus* and the highly frequent pathogen *S. aureus*.

### 4.2. MRSA Biotypes

Considering *S. aureus* species, automated microbiological characterization (MALDI-TOF MS and the Decision Tree method) showed a high frequency of oral clinical isolates of MRSA (60.7%), including strains with SCC*mec* type IV (26.2%), SCC*mec* type II (29.5%), and concomitantly with SCC*mec* type II and IV (7.4%) from 21 patients with orofacial clefts under hospitalization and medical follow-up. Among MRSA-positive patients, the intraoral frequency ranged from 20 to 100%. The time of admission to the operating room (period A) showed a high frequency of MRSA (38.5%), including SCC*mec* type IV (13.1%) and SCC*mec* type II (16.4%). However, lower frequencies of MRSA (14.8%) were detected immediately after surgical rehabilitation (period C) and at the patient’s clinical return after surgery (period D). Patients with orofacial clefts more routinely require health care and clinical examinations in dental centers and hospitals. In part, this high clinical periodicity could explain the high frequency of oral MRSA strains in these patients. Thus, MALDI-TOF MS associated with the Decision Tree method can be considered to simply and quickly characterize MRSA and favorably screen, monitor, and diagnose potential clinical cases involving infections during hospital stays and surgical interventions. As previously reported [72], the preliminary detection of MRSA using MALDI-TOF could optimize microbiological diagnostic time and routine procedures with high predictive value and provide useful information for the management of critically ill patients in hospital settings.

The Decision Tree analyses have shown high sensitivity (96.5%), specificity (73.0%), positive predictive value (90.7%), and negative predictive value (88.5%) when identifying specific peaks for the discrimination of *S. aureus* SCC*mec* types II, III and IV [33]. Although these analyses are potentially capable of differentiating MRSA strains from MSSA, their classification system is limited exclusively to SCC*mec* types II, III, and IV, being applicable only to some MRSA strains. Furthermore, SCC*mec* type IV and MSSA have similar spectral patterns (MALDI-TOF), challenging the potential for discrimination between them [33]. Therefore, the preliminary data of this study reveal some limitations regarding the molecular (e.g., identification of SCC*mec* types I-XIII and subtypes of SCC*mec* elements), epidemiological (e.g., continuous vigilance through monitoring the microbial virulence factors, host specificity, and transmission routes of newer MRSA strains in healthcare professionals and hospital settings, assessment of infection control measures), and therapeutic (e.g., β-lactam or non-β-lactam antibiotics) aspects [73,74], although it signals the importance of monitoring and controlling multidrug-resistant pathogens. In this context, one or more typing methods could be applied, depending on the objective and target to be achieved in the epidemiological and molecular investigations of MRSA, such as pulsed-field gel electrophoresis, multilocus sequence typing, *spa* typing, SCC*mec* typing, microarrays, and whole-genome sequencing [73,74,75,76].

Another important methodological criterion should be the inclusion of type-strains or reference strains as controls in automated microbiological diagnostics (MATDI-TOF) for HA-MRSA and CA-MRSA and their SCC*mec* (sub)types. In this research, *Staphylococcus* species were identified by microbiological methods and MALDI-TOF MS technology, but such controls (i.e., reference SCC*mec* types) were not included. The assays reproducibility was ensured by sampling analysis in triplicate systems, whereas MALDI-TOF assay calibration was carried out by the test standard (Bruker Daltonics) using *Escherichia coli* DH5 alpha, and their Biotyper Classification Results (BCR) displayed (i) secure genus identification, (ii) probable species identification, and/or (iii) highly probable species identification (average score of 2.1718 ± 0.2048).

The mobile genetic element (SCC*mec*) is a determinant for broad-spectrum β-lactam resistance. SCC*mec* types I, II, and III are predominantly found in hospital-acquired MRSA strains (HA-MRSA) and are multidrug-resistant, whereas SCC*mec* types IV and V are most frequently carried by community-associated MRSA strains (CA-MRSA) and tend to be susceptible to most non-β-lactam antibiotics [73,74]. Therefore, knowing the type of SCC*mec* may be important for in-hospital MRSA response strategies [72]. In Latin America, especially in Brazil, colonization and infection cases of CA-MRSA and HA-MRSA isolates and SCC*mec* types (I, II, III, IV, and V) have been reported in some regions. However, epidemiological and molecular studies of *S. aureus* and MRSA are limited in Brazil, which still requires the adoption of assertive government strategies and the implementation of epidemiological surveillance of this pathogen [74].

A rare overlapping effect of SCC*mec* types was also observed in this study. Although the Decision Tree method discriminated between potential MRSA and MSSA clinical strains, it showed an overlapping classification of SCC*mec* types II and IV in nine MRSA isolates. Such isolates showed biotyper classification results (BCR) > 2. At least in part, this overlap could be explained by the variation in sensitivity (SE), specificity (SP), and positive (PPV) and negative (NPV) predictive values of each peak (*m*/*z*) involved in the (i) classification of SCC*mec* type II (peak at 1975 *m*/*z*: SE of 80.6%, SP of 80%, PPV of 79.1%, and NPV of 81.5%; peak at 2592 *m*/*z*: SE of 23.9%, SP of 95.2%, PPV of 82.2%, and NPV of 57.1%; peak at 3890 *m*/*z*: SE of 54.8%, SP of 98.2%, PPV of 96.6%, and NPV of 67.8%); and (ii) classification of SCC*mec* type IV (peak at 5541 *m*/*z*: SE of 75.8%, SP of 90.2%, PPV of 47.2%, and NPV of 97%) [33]. In a recent study [72], the presumptive and accurate identification of MRSA and SCC*mec* types was evaluated by the machine learning-based identification system (AMRQuest software, v.2.1, ASTA: Suwon, Korea) for MALDI-TOF peaks. This system showed high sensitivity (91.8%), specificity (83.3%), and accuracy (87.6%) in distinguishing MRSA, as well as high accuracy (90.9%) for MSSA. For staphylococcal cassette chromosome *mec* types, high accuracy was observed in classifying SCC*mec* II (95.4%) and IVA (96.1%) types, except for SCC*mec* IV (21.4%). These data pointed to the need to include MALDI-TOF spectra involving more type IV clinical isolates in machine model training for better differentiation and MSSA performance [72]. In the molecular context, the SCC*mec* classification into types and subtypes has been based on the high diversity of the structural organization and genetic content of these elements (i.e., *mec* gene complex, *ccr* gene complex, and joining regions). In addition, composite SCC elements (i.e., SCC elements carrying two or more gene complexes) have been identified in various *S. aureus* strains, which must be compared with SCC*mec* types (i.e., determination of the existing SCC*mec* type) and genetically characterized (i.e., combination types and mechanisms of the distinct SCC elements) [73]. However, greater efforts are still needed to understand these genetic and evolutionary phenomena: (i) virulence factors and antimicrobial resistance of *S. aureus*; (ii) clinical symptoms; (iii) therapeutic and hospital management; (iv) epidemiological aspects; and (v) the development of accurate, rapid, and automated methods of microbiological diagnosis. Whether genetic and evolutionary aspects can decrease the specificity and sensitivity of the current MALDI-TOF technology during the identification of *S. aureus* genetic subtypes (e.g., isolates unclassified as MRSA or MSSA, overlapping classification of SCC*mec* types) remains to be determined in future investigations.

The most common complications among patients undergoing cleft repairs are dehiscence and infection [77]. The identification of the microorganisms involved in the infection is of paramount importance in reducing transmission and ensuring that colonized patients are treated [78]. Although MRSA is the main microorganism involved in nosocomial infections, the epidemiology of *S. aureus* is changing and promoting an alert to community-associated MRSA (CA-MRSA) infections [79]. CA-MRSA typically carries a smaller cassette that confers resistance only to b-lactam antibiotics (SCC*mec* type IV, V, or VII) and genes encoding Panton-Valentine Leukocidin (PVL) leukotoxin, lukF/lukS, susceptible to non-β-lactam antimicrobials [80]. Originally, these isolates were reported to be resistant only to methicillin, but later resistance to macrolides and fluoroquinolones arose [81]. CA-MRSA is becoming a worldwide emergency, with some studies reporting the presence of strains in samples from the oropharyngeal region in children, ranging from 7.1% to 0.6% [82,83]. The worldwide spread of PVL-producing MRSA has been reported in several communities. Molecular studies involving clinical isolates of *S. aureus* from Belgian patients revealed PVL-positive strains (lukS-lukF genes) of MRSA acquired in the community. Some patients associated with skin or soft tissue infections, bacteremia, and peritonitis have reported recent travel to North Africa and South America, indicating the emergence and sporadic importation of PVL-positive CA-MRSA clones [84]. In Brazil, rehabilitation centers are located in more developed regions of the country, and patients with craniofacial anomalies who are not residents of these regions need to travel long distances to get access to treatment [85], being able to acquire and disseminate strains associated with the community. A university hospital in Pakistan identified, in a period of 2 years, five cases of CA-MRSA infection in children aged 0.5 months to 11 years old; all of them had an invasive MRSA infection that was finally diagnosed as causing thoracic empyema, infective endocarditis, psoas abscess, and necrotizing fasciitis [86]. CA-MRSA is an emerging pediatric pathogen in Brazil. A study conducted at a tertiary public university hospital in Brazil identified that CA-MRSA was responsible for approximately one-third of all strains isolated from *S. aureus* in children with severe infections, also detecting a trend of increasing incidence density over the five years of the study [87]. The technology of identification and analysis of resistance with MALDI-TOF MS peak profiles can allow faster tests to identify microorganisms, accelerating the evolution of the treatment of multidrug-resistant bacterial infections, thus contributing to the suppression of antimicrobial resistance and reducing the use of broad-spectrum antibiotic therapies [88].

In many countries, the incidence of HA-MRSA is very high in hospitals; that is, the most important classes of antibiotics used to prevent and treat infections are ineffective [89]. The HA-MRSA isolates present in their genome have the mobile genetic elements SCC*mec* type I, II, or III. Strains with SCC*mec* type I have potential resistance to β-lactam antibiotics, due to the *mecA* gene integrated into their genomic sequence, while those with SCC*mec* types II and III may exhibit antimicrobial resistance to multiple classes of antibiotics due to the additional genes integrated into their genomic elements [90]. SCC*mec* type II was first identified in MRSA strains isolated in Japan in 1982 and is usually epidemiologically related to the Asian continent [91]. MRSA strains containing SCC*mec* type II also correspond to genotypes that are known to be related to hospital environments and health professionals [92]. This relationship can be attributed to the presence of ACME type II (arginine catabolic mobile element), which can give the microorganism a better ability to colonize the skin and mucous membranes and, thus, favor its transmission [93]. MRSA strains containing SCC*mec* type II can also carry the *psm-mec* gene in their mobile genetic element, which codes for α-phenol-soluble modulins (PSMs). MRSA with SCC*mec* type II, introducing the *psm-mec* gene intact purportedly suppresses the expression of PSMα, a cytolytic toxin of *S. aureus* involved in a variety of functions, including toxin production, biofilm formation, and colony propagation [94]. A recent study involving virulence genes related to infection by MRSA strains containing SCC*mec* types II and IV revealed that factors related to the bacteriological characteristics and clinical conditions of the host are directly associated with the severity of the infection [95]. Besides these clinical findings, *Staphylococcus* spp. can also show importance in other human contexts [96].

## 5. Conclusions

This research highlights the oral staphylococcal microbiota in patients with orofacial clefts under clinical follow-up and surgical therapy. The population of patients admitted to the operating room shows a low frequency of oral staphylococcal species, ranging from (i) homogeneous colonization by *S. aureus* or *S.* non-*aureus*, (ii) heterogeneous (mixed) colonization by *S. aureus* and *Staphylococcus* spp., and (iii) *S.* non-*aureus* and *Staphylococcus* spp. However, a considerable frequency of *S. aureus* and Staphylococcus spp. (homogeneous and/or heterogeneous colonization) is reported in male patients with CRT or CLT with a history of surgical rehabilitation (cheiloplasty and palatoplasty), regardless of age. Another surprisingly important revelation from the hospital and medical therapeutic aspects is the potential high frequency of MRSA, including HA-MRSA (SCC*mec* type II) and CA-MRSA (SCC*mec* type IV), although these data require confirmation by genomic methods, in patients with *S. aureus*-positive orofacial clefts during hospital admission and prior to surgical asepsis. The highly frequent clinical periodicity in specialized medical and dental centers of these patients may contribute to the acquisition of MRSA.

Antibiotic prophylaxis, asepsis (chlorhexidine or PVPI), and surgical procedures influence the reduction and composition of oral staphylococcal species in rehabilitated patients with orofacial clefts. This dynamic of oral staphylococcal colonization throughout treatment and medical follow-up suffers from the influence of (i) imbalance in aseptic maintenance, (ii) efficiency of surgical asepsis (septic elimination) or break of the aseptic chain (septic neocolonization), (iii) staphylococcal neocolonization in new anatomical habitats of the surgically rehabilitated oral cavity, and (iv) total (septic maintenance) or partial (simultaneous septic elimination and septic maintenance) maintenance of certain staphylococcal oral species.

Finally, the MALDI-TOF MS technology and the Decision Tree method prove to be automated tools for the rapid microbiological diagnosis of MRSA and MSSA, whether in screening, monitoring, or diagnosing potential clinical cases of infections throughout hospital stays and surgical interventions.

## Figures and Tables

**Table 1:** The incidence of oral *Staphylococcus* species isolated from patients with orofacial clefts in period A. The frequency data were subjected to multivariate statistics (SAS^®^ version 9.2): biplots (PCA) and dendrograms (CA) analysis (threshold ≤ 0.5): age group, gender, types of orofacial clefts, surgical history, and types of previous surgical rehabilitation.

Characteristic of Patients with Orofacial Clefts	Presence of *Staphylococcus* Species								Absence of *Staphylococcus* Species		Σ	
	*S. aureus* *		*S. aureus* and *Staphylococcus* Species **		Non-*aureus Staphylococcus* Species ***		Σ					
	*n*	%	*n*	%	*n*	%	*n*	%	*n*	%	*n*	%
Age group												
Infant	2	3.4	0	0.0	3	5.1	5	8.5	8	13.6	13	22.0
Toddler	1	1.7	2	3.4	2	3.4	5	8.5	9	15.3	14	23.7
Preschooler	1	1.7	1	1.7	1	1.7	3	5.1	4	6.8	7	11.9
Childhood	4	6.8	1	1.7	0	0.0	5	8.5	7	11.9	12	20.3
Teenager	1	1.7	0	0.0	1	1.7	2	3.4	1	1.7	3	5.1
Adult	3	5.1	0	0.0	0	0.0	3	5.1	7	11.9	10	16.9
Σ	12	20.3	4	6.8	7	11.9	23	39.0	36	61.0	59	100
Gender												
Female	3	5.1	0	0.0	2	3.4	5	8.5	12	20.3	17	28.8
Male	9	15.3	4	6.8	5	8.5	18	30.5	24	40.7	42	71.2
Σ	12	20.3	4	6.8	7	11.9	23	39.0	36	61.0	59	100
Types of orofacial clefts												
CBT	1	1.7	2	3.4	2	3.4	5	8.5	4	6.8	9	15.3
CRT or CLT	9	15.3	0	0.0	4	6.8	13	22.0	9	15.3	22	37.3
CPo-FC	0	0.0	0	0.0	0	0.0	0	0.0	2	3.4	2	3.4
CPo-FI	0	0.0	0	0.0	0	0.0	0	0.0	4	6.8	4	6.8
CP-FRC or CP-FLC	0	0.0	0	0.0	0	0.0	0	0.0	5	8.5	5	8.5
CP-FRI or CP-FLI	0	0.0	2	3.4	1	1.7	3	5.1	4	6.8	7	11.9
CSPo-FI	1	1.7	0	0.0	0	0.0	1	1.7	0	0.0	1	1.7
DG-G	0	0.0	0	0.0	0	0.0	0	0.0	1	1.7	1	1.7
CP-FRI or CP-FLI + CPo-FI	0	0.0	0	0.0	0	0.0	0	0.0	5	8.5	5	8.5
CRT or CLT + CP-FRI or CP-FLI	1	1.7	0	0.0	0	0.0	1	1.7	2	3.4	3	5.1
Σ	12	20.3	4	6.8	7	11.9	23	39.0	36	61.0	59	100
Surgical history												
Surgical rehabilitation	8	13.6	3	5.1	4	6.8	15	25.4	20	33.9	35	59.3
No surgical rehabilitation	4	6.8	1	1.7	3	5.1	8	13.6	16	27.1	24	40.7
Σ	12	20.3	4	6.8	7	11.9	23	39.0	36	61.0	59	100
Types of previous surgical rehabilitation												
Che	3	5.1	1	1.7	2	3.4	6	10.2	8	13.6	14	40.0
Pal	1	1.7	0	0.0	0	0.0	1	1.7	2	3.4	3	8.6
Rhi	0	0.0	0	0.0	0	0.0	0	0.0	0	0.0	0	0.0
Che and Pal	2	3.4	2	3.4	1	1.7	5	8.5	7	11.9	12	34.3
Che and Rhi	0	0.0	0	0.0	0	0.0	0	0.0	2	3.4	2	5.7
Pal and Rhi	1	1.7	0	0.0	0	0.0	1	1.7	0	0.0	1	2.9
Che, Pal and Rhi	1	1.7	0	0.0	1	1.7	2	3.4	1	1.7	3	8.6
Σ	8	13.6	3	5.1	4	6.8	15	25.4	20	33.9	35	100

Infant (from birth to 1 year old), toddler (1–3 years), preschooler (3–5 years), childhood (6–11 years), teenager (12–17 years), and adult (over 18 years). Cleft Bilateral Transforaminal (CBT), Cleft Right or Left Transforamen (CRT or CLT), Cleft Post-Foramen Complete (CPo-FC), Cleft Post-Foramen Incomplete (CPo-FI), Cleft Pre-Foramen Right or Left Complete (CP-FRC or CP-FLC), Cleft Pre-Foramen Right or Left Incomplete (CP-FRI or CP-FLI), Cleft Submucosa Post-Foramen Incomplete (CSPo-FI), Deformity Groove-Gingival (DG-G), Cleft Pre-Foramen Right or Left Incomplete (CP-FRI or CP-FLI) and Cleft Post-Foramen Incomplete (CPo-FI), and Cleft Right or Left Transforamen (CRT or CLT) and Cleft Pre-Foramen Right or Left Incomplete (CP-FRI or CP-FLI). Che (cheiloplasty), Pal (palatoplasty), and Rhi (rhinoseptoplasty). * Patient colonized by a single species of the genus and defined as homogeneous colonization, that is, *S. aureus*. ** Patient colonized by two or more species of the genus *Staphylococcus* and defined as heterogeneous colonization, including *S. aureus*. *** Patient colonized by one or more species of the genus *Staphylococcus* and can be defined as homogeneous or heterogeneous colonization, excluding *S. aureus*.

**Table 2:** The incidence of oral *Staphylococcus* species isolated from patients with orofacial clefts throughout the pre- and post-surgical periods (periods A, B, C, and D). The frequency data were subjected to multivariate statistics (SAS^®^ version 9.2): biplots (PCA) and dendrograms (CA) analysis (threshold ≤ 0.5). Epidemiological variable: “periods of hospitalization” and “surgical asepsis procedures”.

Microbiological Characteristics	Period A		Period B		Period C		Period D		∑	
	*n*	%	*n*	%	*n*	%	*n*	%	*n*	%
Group chlorhexidine										
*S. aureus* *	9	19.6	0	0.0	4	8.7	3	10.0	16	9.5
*S. aureus* and *Staphylococcus* species **	2	4.3	0	0.0	2	4.3	0	0.0	4	2.4
non-*aureus Staphylococcus* species ***	6	13.0	2	4.3	2	4.3	1	3.3	11	6.5
∑ (*Staphylococcus* species)	17	37.0	2	4.3	8	17.4	4	13.3	31	18.5
Absence of *Staphylococcus* species	29	63.0	44	95.7	38	82.6	26	86.7	137	81.5
∑ (presence and absence)	46	100.0	46	100.0	46	100.0	30	100.0	168	100.0
Group PVPI										
*S. aureus* *	3	23.1	0	0.0	2	15.4	3	27.3	8	16.0
*S. aureus* and *Staphylococcus* species **	2	15.4	0	0.0	0	0.0	0	0.0	2	4.0
non-*aureus Staphylococcus* species ***	1	7.7	0	0.0	0	0.0	0	0.0	1	2.0
∑ (*Staphylococcus* species)	6	46.2	0	0.0	2	15.4	3	27.3	11	22.0
Absence of *Staphylococcus* species	7	53.8	13	100.0	11	84.6	8	72.7	39	78.0
∑ (presence and absence)	13	100.0	13	100.0	13	100.0	11	100.0	50	100.0
Groups chlorhexidine and PVPI										
*S. aureus* *	12	20.3	0	0.0	6	10.2	6	14.6	24	11.0
*S. aureus* and *Staphylococcus* species **	4	6.8	0	0.0	2	3.4	0	0.0	6	2.8
non-*aureus Staphylococcus* species ***	7	11.9	2	3.4	2	3.4	1	2.4	12	5.5
∑ (*Staphylococcus* species)	23	39.0	2	3.4	10	16.9	7	17.1	42	19.3
Absence of *Staphylococcus* species	36	61.0	57	96.6	49	83.1	34	82.9	176	80.7
∑ (presence and absence)	59	100.0	59	100.0	59	100.0	41	100.0	218	100.0

Period A: admission to surgical ward prior to asepsis; Period B: prior to surgical procedure and immediately after asepsis with PVPI or chlorhexidine; Period C: immediately after surgical rehabilitation; and Period D: at the first patient return to the Centro Pró-Sorriso aos Portadores de Fissuras Labial e Palatina, Alfenas, MG, Brazil. * Patient colonized by a single species of the genus and defined as homogeneous colonization, that is, *S. aureus*. ** Patient colonized by two or more species of the genus *Staphylococcus* and defined as heterogeneous colonization, including *S. aureus*. *** Patient colonized by one or more species of the genus *Staphylococcus* and can be defined as homogeneous or heterogeneous colonization, excluding *S. aureus*.

**Table 3:** Dynamics of oral colonization by *Staphylococcus* species isolated from patients with orofacial clefts throughout the pre- and post-surgical periods (periods A, B, C, and D). The frequency data were subjected to multivariate statistics (SAS^®^ version 9.2): biplots (PCA) and dendrograms (CA) analysis (threshold ≤ 0.5).

Colonization Dynamics	Period A		Period B		Period C		Period D		∑	
	*n*	%	*n*	%	*n*	%	*n*	%	*n*	%
Group chlorhexidine										
Aseptic maintenance (AM)	29	63.0	27	58.7	24	52.2	14	46.7	94	56.0
Septic elimination (SE)	0	0.0	17	37.0	14	30.4	12	40.0	43	25.6
Septic neocolonization (SN)	0	0.0	2	4.3	5	10.9	4	13.3	11	6.5
Septic maintenance (SM)	17	37.0	0	0.0	1	2.2	0	0.0	18	10.7
SE and SN simultaneously	0	0.0	0	0.0	2	4.3	0	0.0	2	1.2
SE and SM simultaneously	0	0.0	0	0.0	0	0.0	0	0.0	0	0.0
∑	46	100.0	46	100.0	46	100.0	30	100.0	168	100.0
Group PVPI										
Aseptic maintenance (AM)	7	53.8	7	53.8	6	46.2	6	54.5	26	52.0
Septic elimination (SE)	0	0.0	6	46.2	5	38.5	2	18.2	13	26.0
Septic neocolonization (SN)	0	0.0	0	0.0	1	7.7	1	9.1	2	4.0
Septic maintenance (SM)	6	46.2	0	0.0	1	7.7	1	9.1	8	16.0
SE and SN simultaneously	0	0.0	0	0.0	0	0.0	0	0.0	0	0.0
SE and SM simultaneously	0	0.0	0	0.0	0	0.0	1	9.1	1	2.0
∑	13	100.0	13	100.0	13	100.0	11	100.0	50	100.0
Groups chlorhexidine and PVPI										
Aseptic maintenance (AM)	36	61.0	34	57.6	30	50.8	20	48.8	120	55.0
Septic elimination (SE)	0	0.0	23	39.0	19	32.2	14	34.1	56	25.7
Septic neocolonization (SN)	0	0.0	2	3.4	6	10.2	5	12.2	13	6.0
Septic maintenance (SM)	23	39.0	0	0.0	2	3.4	1	2.4	26	11.9
SE and SN simultaneously	0	0.0	0	0.0	2	3.4	0	0.0	2	0.9
SE and SM simultaneously	0	0.0	0	0.0	0	0.0	1	2.4	1	0.5
∑	59	100.0	59	100.0	59	100.0	41	100.0	218	100.0

Period A: admission to surgical ward prior to asepsis; Period B: prior to surgical procedure and immediately after asepsis with PVPI or chlorhexidine; Period C: immediately after surgical rehabilitation; and Period D: at the first patient return to the Centro Pró-Sorriso aos Portadores de Fissuras Labial e Palatina, Alfenas, MG, Brazil. AM (aseptic maintenance), SE (septic elimination), SN (septic neocolonization), and SM (septic maintenance).

**Table 4:** Frequency and classification of clinical species of MRSA (methicillin-resistant *Staphylococcus aureus*) and MSSA (methicillin-sensitive *Staphylococcus aureus*) isolated from patients with orofacial clefts during the admission period to the surgical center (prior to asepsis: period A), immediately after surgical rehabilitation (period C), and return of the patient after surgery (period D), using the MALDI-TOF MS technology and Decision Tree method.

CP	P	CI	Peaks (*m*/*z*)											MRSA					MSSA	U
			1975	2194	2339	2410	2592	2631	3890	4607	5053	5541	6594	N1	N2	N3	N3	∑	N4	N5
														IV	III		II			
CLP1	A	8	-	-	-	-	-	-	-	4607	5053	-	-	-	-	1	-	1	-	-
CLP1	A	9	-	-	-	-	-	-	3890	4607	5053	-	-	-	-	-	1	1	-	-
CLP1	A	10	-	-	-	-	-	-	3890	-	-	-	-	-	-	-	1	1	-	-
CLP1	A	11	-	-	-	-	-	-	3890	4607	5053	-	-	-	-	-	1	1	-	-
CLP1	A	78	-	-	-	-	-	-	-	-	-	-	6594	-	-	1	-	1	-	-
CLP1	A	79	-	-	-	-	-	-	-	-	-	-	6594	-	-	1	-	1	-	-
CLP1	A	80	-	-	-	-	-	-	-	-	-	-	6594	-	-	1	-	1	-	-
CLP1	A	81	-	-	-	2410	-	-	-	-	-	5541	-	1	-	-	-	1	-	-
CLP1	A	82	-	-	-	-	-	-	-	-	-	-	6594	-	-	1	-	1	-	-
													∑(*n*)	1	0	5	3	9	0	0
													%	11.1	0.0	55.6	33.3	100.0	0.0	0.0
CLP4	A	42	-	-	-	-	-	2631	3890	-	5053	5541	6594	-	-	-	-	0	-	1
CLP4	A	43	-	-	-	-	-	-	-	-	5053	5541	-	-	-	-	-	0	1	-
CLP4	A	44	-	-	-	-	-	-	-	-	5053	5541	-	-	-	-	-	0	1	-
CLP4	A	45	-	-	-	-	-	-	3890	-	5053	5541	-	-	-	-	1	1	-	-
CLP4	D	29	-	-	-	-	-	-	-	-	-	5541	-	1	-	-	-	1	-	-
CLP4	D	30	-	-	-	-	-	-	-	-	-	5541	6594	1	-	-	-	1	-	-
CLP4	D	31	-	-	-	-	-	-	-	-	-	5541	-	1	-	-	-	1	-	-
CLP4	D	32	-	-	-	-	-	-	-	-	-	5541	-	1	-	-	-	1	-	-
CLP4	D	33	-	-	-	-	-	-	-	-	-	5541	-	1	-	-	-	1	-	-
													∑(*n*)	5	0	0	1	6	2	1
													%	55.6	0.0	0.0	11.1	66.7	22.2	11.1
CLP7	A	12	-	-	-	-	-	-	-	-	5053	5541	-	-	-	-	-	0	1	-
CLP7	A	13	-	-	-	-	-	-	-	-	5053	5541	6594	-	-	1	-	1	-	-
CLP7	A	14	-	-	-	-	-	-	-	-	5053	5541	-	-	-	-	-	0	1	-
CLP7	A	15	-	-	-	-	-	-	-	-	5053	-	-	-	-	-	-	0	1	-
CLP7	A	16	-	-	-	2410	-	-	-	-	5053	-	6594	-	-	1	-	1	-	-
CLP7	C	22	-	-	-	-	-	-	-	-	5053	-	-	-	-	-	-	0	1	-
CLP7	C	23	-	-	-	-	-	2631	-	-	5053	5541	-	-	-	-	-	0	1	-
CLP7	C	24	-	-	-	-	-	-	-	-	-	-	-	-	-	-	-	0	-	1
CLP7	C	25	-	-	-	-	-	-	-	-	-	5541	-	1	-	-	-	1	-	-
CLP7	C	26	-	-	-	-	-	-	-	-	5053	5541	-	-	-	-	-	0	1	-
CLP7	D	97	-	2194	2339	-	-	2631	3890	-	-	-	6594	-	-	-	-	0	-	1
CLP7	D	98	-	-	-	-	-	2631	3890	-	5053	-	6594	-	-	-	-	0	-	1
													∑(*n*)	1	0	2	0	3	6	3
													%	8.3	0.0	16.7	0.0	25.0	50.0	25.0
CLP8	A	17	-	-	-	-	-	-	3890	-	5053	-	6594	-	-	-	1	1	-	-
CLP8	A	18	-	-	-	-	-	-	-	-	-	5541	-	1	-	-	-	1	-	-
CLP8	A	19	1975	2194	2339	2410	2592	2631	3890	-	5053	5541	-	-	-	-	-	0	-	1
CLP8	A	20	-	-	-	-	-	-	-	-	-	5541	6594	1	-	-	-	1	-	-
CLP8	A	21	-	-	-	-	-	-	-	-	-	5541	-	1	-	-	-	1	-	-
													∑(*n*)	3	0	0	1	4	0	1
													%	60.0	0.0	0.0	20.0	80.0	0.0	20.0
CLP9	A	34	-	-	-	-	-	-	3890	-	-	5541	6594	1	-	-	1	1 *	-	-
CLP9	A	35	-	-	-	-	-	-	3890	-	-	5541	6594	1	-	-	1	1 *	-	-
CLP9	A	36	-	-	-	-	-	-	3890	-	5053	5541	-	-	-	-	1	1	-	-
CLP9	A	37	1975	-	-	2410	-	-	3890	-	5053	5541	6594	-	-	-	1	1	-	-
CLP9	A	117	-	-	-	-	-	-	3890	4607	5053	-	-	-	-	-	1	1	-	-
													∑(*n*)	2	0	0	5	5	0	0
													%	40.0	0.0	0.0	100.0	100.0	0.0	0.0
CLP10	C	105	-	2194	-	-	-	-	3890	-	5053	5541	6594	-	-	-	-	0	-	1
													∑(*n*)	0	0	0	0	0	0	1
													%	0.0	0.0	0.0	0.0	0.0	0.0	100.0
CLP15	C	27	-	-	-	-	-	-	-	-	-	5541	-	1	-	-	-	1	-	-
CLP15	C	28	-	-	-	-	-	-	3890	-	5053	5541	6594	-	-	-	1	1	-	-
CLP15	C	99	-	2194	2339	-	-	2631	3890	-	-	-	-	-	-	-	-	0	-	1
CLP15	C	100	-	-	-	-	-	-	-	-	-	5541	6594	1	-	-	-	1	-	-
CLP15	D	101	-	-	-	-	-	2631	-	-	5053	5541	-	-	-	-	-	0	1	-
CLP15	D	102	-	2194	2339	-	-	2631	3890	-	5053	5541	6594	-	-	-	-	0	-	1
CLP15	D	103	-	-	-	-	-	2631	3890	-	5053	5541	6594	-	-	-	-	0	-	1
CLP15	D	115	-	2194	2339	-	-	2631	-	-	-	-	-	-	-	-	-	0	1	-
CLP15	D	121	-	-	-	-	-	-	-	-	-	5541	-	1	-	-	-	1	-	-
													∑(*n*)	3	0	0	1	4	2	3
													%	33.3	0.0	0.0	11.1	44.4	22.2	33.3
CLP18	C	104	1975	2194	-	-	2592	2631	3890	-	5053	5541	-	-	-	-	-	0	-	1
													∑(*n*)	0	0	0	0	0	0	1
													%	0.0	0.0	0.0	0.0	0.0	0.0	100.0
CLP23	A	53	-	-	-	-	-	-	3890	-	-	5541	6594	1	-	-	1	1 *	-	-
CLP23	A	54	-	-	-	-	2592	-	-	-	-	5541	6594	1	-	-	1	1 *	-	-
CLP23	A	55	-	-	-	-	2592	-	-	-	-	5541	6594	1	-	-	1	1 *	-	-
CLP23	A	56	-	-	-	-	2592	-	-	-	-	5541	-	1	-	-	1	1 *	-	-
CLP23	A	57	1975	2194	2339	2410	2592	2631	-	-	5053	5541	-	-	-	-	-	0	-	1
													∑(*n*)	4	0	0	4	4	0	1
													%	80.0	0.0	0.0	80.0	80.0	0.0	20.0
CLP24	C	6	-	-	-	-	-	-	-	-	-	-	6594	-	-	1	-	1	-	-
CLP24	C	7	-	-	2339	-	-	2631	-	-	5053	-	-	-	-	-	-	0	1	-
													∑(*n*)	0	0	1	0	1	1	0
													%	0.0	0.0	50.0	0.0	50.0	50.0	0.0
CLP25	C	48	-	-	-	-	-	-	-	-	-	5541	-	1	-	-	-	1	-	-
CLP25	C	49	-	-	-	-	-	-	-	-	5053	5541	-	-	-	-	-	0	1	-
CLP25	C	50	-	-	-	-	-	-	-	-	-	5541	-	1	-	-	-	1	-	-
CLP25	C	51	-	-	-	-	2592	-	-	-	-	5541	-	1	-	-	1	1*	-	-
CLP25	C	52	-	-	-	-	-	-	-	-	-	5541	6594	1	-	-	-	1	-	-
CLP25	C	93	-	-	-	-	-	-	3890	-	5053	-	6594	-	-	-	1	1	-	-
CLP25	C	94	1975	2194	2339	2410	2592	2631	3890	-	-	-	6594	-	-	-	-	0	-	1
CLP25	C	95	-	-	-	-	-	2631	3890	-	-	-	6594	-	-	-	-	0	-	1
CLP25	C	96	-	-	-	-	-	2631	3890	-	-	-	6594	-	-	-	-	0	-	1
CLP25	C	118	-	2194	2339	-	-	2631	-	-	-	-	-	-	-	-	-	0	1	-
CLP25	D	1	-	-	-	-	-	-	-	-	5053	5541	-	-	-	-	-	0	1	-
CLP25	D	2	-	-	-	-	-	-	3890	-	5053	5541	-	-	-	-	1	1	-	-
CLP25	D	3	-	-	-	-	-	-	3890	-	-	5541	-	1	-	-	1	1 *	-	-
CLP25	D	4	-	-	-	-	-	-	-	-	-	5541	-	1	-	-	-	1	-	-
CLP25	D	5	-	-	-	-	-	-	-	-	-	5541	-	1	-	-	-	1	-	-
													∑(*n*)	7	0	0	4	9	3	3
													%	46.7	0.0	0.0	26.7	60.0	20.0	20.0
CLP30	A	116	1975	2194	-	2410	2592	2631	-	-	5053	-	-	-	-	-	-	0	-	1
													∑(*n*)	0	0	0	0	0	0	1
													%	0.0	0.0	0.0	0.0	0.0	0.0	100.0
CLP36	A	111	-	-	-	-	-	-	3890	-	5053	-	6594	-	-	-	1	1	-	-
CLP36	A	112	-	-	-	-	-	-	3890	-	5053	5541	6594	-	-	-	1	1	-	-
CLP36	A	120	1975	2194	-	2410	2592	2631	-	-	5053	5541	-	-	-	-	-	0	-	1
													∑(*n*)	0	0	0	2	2	0	1
													%	0.0	0.0	0.0	66.7	66.7	0.0	33.3
CLP37	A	38	-	-	-	-	-	-	-	-	-	5541	6594	1	-	-	-	1	-	-
CLP37	A	39	-	-	-	-	-	-	-	-	-	5541	6594	1	-	-	-	1	-	-
CLP37	A	40	1975	2194	2339	2410	2592	2631	-	-	5053	5541	-	-	-	-	-	0	-	1
CLP37	A	41	-	-	-	-	-	-	3890	-	-	5541	6594	1	-	-	1	1 *	-	-
CLP37	C	106	-	-	-	-	-	-	3890	4607	5053	5541	6594	-	-	-	1	1	-	-
CLP37	C	107	-	-	-	-	-	-	3890	-	5053	-	6594	-	-	-	1	1	-	-
CLP37	C	108	-	-	-	-	-	-	3890	-	5053	-	6594	-	-	-	1	1	-	-
CLP37	C	109	-	-	-	-	-	-	3890	-	5053	-	6594	-	-	-	1	1	-	-
CLP37	C	110	-	-	-	-	-	-	3890	-	5053	-	6594	-	-	-	1	1	-	-
													∑(*n*)	3	0	0	6	8	0	1
													%	33.3	0.0	0.0	66.7	88.9	0.0	11.1
CLP40	A	88	-	-	-	-	-	2631	-	-	5053	5541	6594	-	-	-	-	0	-	1
CLP40	A	89	-	2194	2339	-	-	2631	-	-	-	-	6594	-	-	-	-	0	-	1
CLP40	A	90	-	2194	-	-	-	2631	-	-	5053	-	6594	-	-	-	-	0	-	1
CLP40	A	91	-	2194	-	-	-	2631	-	-	5053	-	6594	-	-	-	-	0	-	1
CLP40	A	92	-	-	-	-	-	-	-	-	-	5541	6594	1	-	-	-	1	-	-
													∑(*n*)	1	0	0	0	1	0	4
													%	20.0	0.0	0.0	0.0	20.0	0.0	80.0
CLP48	A	62	-	-	-	-	-	-	-	-	5053	5541	-	-	-	-	-	0	1	-
CLP48	A	63	-	-	-	-	-	-	-	-	5053	5541	-	-	-	-	-	0	1	-
CLP48	A	64	-	-	-	-	-	-	-	-	5053	5541	6594	-	-	1	-	1	-	-
CLP48	A	65	-	-	-	-	-	-	-	-	5053	5541	6594	-	-	1	-	1	-	-
CLP48	A	114	-	-	-	-	-	-	3890	-	5053	5541	-	-	-	-	1	1	-	-
													∑(*n*)	0	0	2	1	3	2	0
													%	0.0	0.0	40.0	20.0	60.0	40.0	0.0
CLP50	A	122	-	-	-	-	-	-	-	-	5053	-	6594	-	-	1	-	1	-	-
													∑(*n*)	0	0	1	0	1	0	0
													%	0.0	0.0	100.0	0.0	100.0	0.0	0.0
CLP51	A	58	-	-	-	-	-	-	-	-	-	5541	-	1	-	-	-	1	-	-
CLP51	A	59	-	-	-	-	-	-	-	-	5053	5541	-	-	-	-	-	0	1	-
CLP51	A	60	-	-	-	-	-	-	-	-	5053	5541	-	-	-	-	-	0	1	-
CLP51	A	61	1975	-	-	-	-	-	-	-	5053	5541	6594	-	-	-	1	1	-	-
CLP51	A	119	-	-	-	-	-	2631	-	-	5053	5541	-	-	-	-	-	0	1	-
													∑(*n*)	1	0	0	1	2	3	0
													%	20.0	0.0	0.0	20.0	40.0	60.0	0.0
CLP54	C	73	-	2194	2339	-	-	2631	-	-	5053	5541	6594	-	-	-	-	0	-	1
CLP54	C	74	-	-	-	-	-	-	3890	-	5053	5541	6594	-	-	-	1	1	-	-
CLP54	C	75	-	-	-	-	-	-	-	-	5053	5541	6594	-	-	1	-	1	-	-
CLP54	C	76	-	-	-	-	-	-	-	-	5053	5541	-	-	-	-	-	0	1	-
CLP54	C	77	-	2194	2339	-	-	2631	-	-	5053	-	-	-	-	-	-	0	1	-
													∑(*n*)	0	0	1	1	2	2	1
													%	0.0	0.0	20.0	20.0	40.0	40.0	20.0
CLP55	D	66	-	-	-	-	-	-	-	-	5053	5541	6594	-	-	1	-	1	-	-
CLP55	D	67	-	-	-	-	-	-	-	-	5053	5541	6594	-	-	1	-	1	-	-
													∑(*n*)	0	0	2	0	2	0	0
													%	0.0	0.0	100.0	0.0	100.0	0.0	0.0
CLP56	A	68	-	2194	-	-	-	2631	-	-	5053	5541	-	-	-	-	-	0	1	-
CLP56	A	69	-	-	-	-	-	-	-	-	5053	5541	-	-	-	-	-	0	1	-
CLP56	A	70	-	-	-	-	-	2631	-	-	5053	5541	-	-	-	-	-	0	1	-
CLP56	A	71	-	-	-	-	-	-	-	-	5053	5541	-	-	-	-	-	0	1	-
CLP56	A	72	-	-	-	-	-	-	-	-	-	5541	6594	1	-	-	-	1	-	-
													∑(*n*)	1	0	0	0	1	4	0
													%	20.0	0.0	0.0	0.0	20.0	80.0	0.0
CLP57	D	83	-	-	-	-	-	-	3890	-	-	-	6594	-	-	-	1	1	-	-
CLP57	D	84	-	-	-	-	-	-	3890	-	5053	-	6594	-	-	-	1	1	-	-
CLP57	D	85	-	-	-	-	-	-	3890	-	5053	5541	6594	-	-	-	1	1	-	-
CLP57	D	86	-	-	-	-	-	-	3890	-	5053	-	6594	-	-	-	1	1	-	-
CLP57	D	87	-	-	-	-	-	-	3890	-	5053	-	6594	-	-	-	1	1	-	-
													∑(*n*)	0	0	0	5	5	0	0
													%	0.0	0.0	0.0	100.0	100.0	0.0	0.0
CLP58	A	113	-	-	-	-	-	-	3890	-	5053	-	6594	-	-	-	1	1	-	-
													∑(*n*)	0	0	0	1	1	0	0
													%	0.0	0.0	0.0	100.0	100.0	0.0	0.0
CLP59	A	46	-	-	-	-	-	2631	-	-	5053	5541	6594	-	-	-	-	0	-	1
CLP59	A	47	-	-	-	-	-	-	-	-	5053	5541	6594	-	-	1	-	1	-	-
													∑(*n*)	0	0	1	0	1	0	1
													%	0.0	0.0	50.0	0.0	50.0	0.0	50.0
∑			Peaks (*m*/*z*)											MRSA					MSSA	U
			1975	2194	2339	2410	2592	2631	3890	4607	5053	5541	6594	N1	N2	N3	N3	∑	N4	N5
														IV	III		II			
*n*			9	19	13	9	11	30	43	5	74	81	62	32	0	15	36	74	25	23
%			7.4	15.6	10.7	7.4	9.0	24.6	35.2	4.1	60.7	66.4	50.8	26.2	0.0	12.3	29.5	60.7	20.5	18.9

* Isolates displaying two classifications (SCC*mec* type II and IV). CP (code of patients), P (period), CI (code of isolate), Nn (node), and U (unclassified). MRSA: Node 1 (SCC*mec* type IV: presence of peak at 5541 *m*/*z* and absence of peak at 5053 *m*/*z*), Node 2 (SCC*mec* type III: concomitant presence of peaks at 2410 and 4607 *m*/*z*), Node 3 (presence of one or more R-peaks: 1975, 2410, 2592, 3890, 4607, or 6594 *m*/*z*; associated with the absence of the S-peaks: 2194, 2339, and 2631 *m*/*z*; SCC*mec* type II: presence of peaks at 1975, 2592, or 3890 *m*/*z*). MSSA: Node 4 (absence of the R-peaks). Unclassified: Node 5 (concomitant presence of one or more R- and S-peaks).

## Data Availability

Dataset available on request from the authors.

## Supplementary Materials

The following supporting information can be downloaded at: https://www.mdpi.com/article/10.3390/pathogens13090763/s1, Table S1: Incidence of oral *Staphylococcus* species isolated from patients with orofacial clefts during admission to surgical ward prior to asepsis (period A), prior to surgical procedure and immediately after asepsis with PVP-I or chlorhexidine (period B), immediately after surgical rehabilitation (period C) and at the first patient return to the *Centro Pró-Sorriso aos Portadores de Fissuras Labial e Palatina*, Alfenas, MG, Brazil (period D: ≥5 and ≤183 days, mean of 54.2 ± 37.6 days); Table S2: Dynamics and incidence of oral colonization by *Staphylococcus* species isolated from patients with orofacial clefts throughout the pre- and post-surgical periods (period A: admission to surgical ward prior to asepsis; period B: prior to surgical procedure and immediately after asepsis with PVP-I or chlorhexidine; period C: immediately after surgical rehabilitation; and period D: at the first patient return to the *Centro Pró-Sorriso aos Portadores de Fissuras Labial e Palatina*, Alfenas, MG, Brazil).
