# Supplementary material for: Oral Staphylococcus Species and MRSA Strains in Patients with Orofacial Clefts Undergoing Surgical Rehabilitation Diagnosed by MALDI-TOF MS
Source: Pathogens. 2024 Sep 5;13(9):763. doi: 10.3390/pathogens13090763 (PMC11434827; doi:10.3390/pathogens13090763)
Supplement: Supplementary file 1 [file pathogens-13-00763-s001.zip › Table S1.pdf]

Supplementary Material – Table S1. Incidence of oral *Staphylococcus* species isolated from patients with orofacial clefts during admission to surgical ward prior to asepsis (period A), prior to surgical procedure and immediately after asepsis with PVP-I or chlorhexidine (period B), immediately after surgical rehabilitation (period C) and at the first patient return to the *Centro Pró-Sorriso aos Portadores de Fissuras Labial e Palatina*, Alfenas, MG, Brazil (period D: ≥5 and ≤183 days, mean of 54.2 ±37.6 days).

**Classification of cleft lip and palate and patient code**	**Isolation of oral *Staphylococcus* species***				**Days**
	**Period A**	**Period B**	**Period C**	**Period D**	**A-D**
**Cleft bilateral transforaminal (CBT) — Female**					
CLP20 (17 years; Che, Pal and Rhi)	*S. saprophyticus*	-	-	∅	-
CLP26 (58 years; Che, Pal and Rhi)	-	-	-	-	22
**Cleft bilateral transforaminal (CBT) — Male**					
CLP1 (3 years; Che and Pal)	*S. aureus* and *S. haemolyticus*	-	-	-	18
CLP4 (2 years; Che and Pal)	*S. aureus* and *S. sciuri*	-	-	*S. aureus*	22
CLP9 (11 years; Che)	*S. aureus*	-	-	∅	-
CLP19 (9 years; Che and Pal)	-	-	-	-	83
CLP27 (11 months)	*S. sciuri*	-	-	-	32
CLP28 (34 years)	-	-	-	-	33
CLP55 (16 years; Che and Pal)	-	-	-	*S. aureus*	33
**Cleft right or left transforamen (CRT or CLT) — Female**					
CLP5 (42 years; Che): CLT	-	-	-	-	26
CLP23 (30 years; Pal and Rhi): CLT	*S. aureus*	-	*S. epidermidis*	∅	-
CLP40 (10 years): CLT	*S. aureus*	-	-	-	85
CLP54 (2 year; Che): CRT	*S. arlettae* and *S. saprophyticus*	-	*S. aureus*	∅	-
**Cleft right or left transforamen (CRT or CLT) — Male**					
CLP7 (45 years; Che): CLT	*S. aureus*	-	*S. aureus*	*S. aureus*	33
CLP8 (30 years; Pal): CLT	*S. aureus*	-	-	-	16
CLP10 (4 months): CLT	-	-	*S. aureus* and *S. epidermidis*	-	123

CLP13 (10 years; Che and Pal): CRT	-	-	-	∅	-
CLP15 (1 year; Che): CRT	-	-	*S. aureus*	*S. aureus*	110
CLP18 (7 months): CRT	-	-	*S. aureus*	-	89
CLP21 (4 months): CRT	-	-	-	-	36
CLP24 (5 months): CLT	-	-	*S. aureus* and *S. epidermidis*	-	68
CLP29 (9 months): CRT	*S. epidermidis*, *S. saprophyticus* and *S. warneri*	-	-	-	5
CLP36 (3 years; Che and Pal): CLT	*S. aureus*	-	-	-	32
CLP37 (11 years; Che and Pal): CRT	*S. aureus*	-	*S. aureus*	-	29
CLP38 (7 years; Che and Pal): CLT	-	-	-	∅	-
CLP43 (1 year; Che): CRT	*S. epidermidis*	-	-	∅	-
CLP44 (3 years; Che and Pal): CRT	*S. epidermidis*	-	-	-	53
CLP48 (3 months): CLT	*S. aureus*	-	-	-	60
CLP51 (1 year; Che): CLT	*S. aureus*	-	-	-	78
CLP56 (15 years; Che, Pal and Rhi): CRT	*S. aureus*	-	-	-	33
CLP57 (1 year; Che): CRT	-	-	-	*S. aureus*	68
**Cleft post-foramen complete (CPo-FC) — Female**					
CLP16 (58 years)	-	*S. saprophyticus*		*S. saprophyticus*	81
**Cleft post-foramen complete (CPo-FC) — Male**					
CLP53 (1 year)	-	-	-	-	68
**Cleft post-foramen incomplete (CPo-FI) — Female**					
CLP14 (8 years; Pal)	-	-	-	∅	-
CLP41 (4 years; Pal)	-	-	-	∅	-
**Cleft post-foramen incomplete (CPo-FI) — Male**					
CLP12 (1 year)	-	-	-	-	110
CLP22 (6 years)	-	-	-	-	29
**Cleft pre-foramen right or left complete (CP-FRC or CP-FLC) — Female**					
CLP17 (11 years): CP-FLC	-	-	-	∅	-
CLP31 (19 years: Che and Rhi): CP-FRC	-	-	-	∅	-

CLP46 (4 years; Che): CP-FRC	-	-	-	-	61
CLP52 (2 year; Che): CP-FLC	-	-	-	∅	-
**Cleft pre-foramen right or left complete (CP-FRC or CP-FLC) — Male**					
CLP6 (3 years; Che): CP-FLC	-	-	-	-	22
**Cleft pre-foramen right or left incomplete (CP-FRI or CP-FLI) — Female**					
CLP35 (1 year; Che): CP-FLI	-	-	-	-	29
**Cleft pre-foramen right or left incomplete (CP-FRI or CP-FLI) — Male**					
CLP2 (5 months): CP-FLI	-	-	-	-	32
CLP25 (30 years; Che and Rhi): CP-FLI	-	-	*S. aureus*	*S. aureus*	39
CLP30 (10 years): CP-FRI	*S. aureus* and *S. saprophyticus*	-	-	∅	-
CLP47 (6 months): CP-FLI	-	-	-	-	61
CLP49 (3 months): CP-FLI	*S. epidermidis*	-	-	-	60
CLP59 (1 year; Che): CP-FLI	*S. aureus* and *S. haemolyticus*	-	-	∅	-
**Cleft submucosa post-foramen incomplete (CSPo-FI) — Female**					
CLP58 (10 years)	*S. aureus*	-	-	-	29
**Deformity groove-gingival (DG-G) — Female**					
CLP3 (9 months)	-	-	-	-	26
**Cleft pre-foramen right or left incomplete (CP-FRI or CP-FLI) and cleft post-foramen incomplete (CPo-FI) — Female**					
CLP33 (1 year): CP-FLI and CPo-FI	-	*S. saprophyticus*	-	∅	-
**Cleft pre-foramen right or left incomplete (CP-FRI or CP-FLI) and cleft post-foramen incomplete (CPo-FI) — Male**					
CLP11 (2 years; Che): CP-FLI and CPo-FI	-	-	-	-	183
CLP32 (42 years): CP-FLI and CPo-FI	-	-	-	-	33
CLP34 (4 years; Che and Pal): CP-FRI and CPo-FI	-	-	-	∅	-
CLP42 (2 years; Che and Pal): CP-FRI and CPo-FI	-	-	-	∅	-
**Cleft right or left transforamen (CRT or CLT) and cleft pre-foramen right or left incomplete (CP-FRI or CP-FLI) — Male**					
CLP39 (9 years; Che and Pal): CLT and CP-FRI	-	-	-	∅	-
CLP45 (10 months): CRT and CP-FLI	-	-	*S. lentus*	-	137
CLP50 (4 months): CLT and CP-FRI	*S. aureus*	-	-	-	36

* Isolation and presumptive identification of *Staphylococcus* species: MSA selective culture medium (Mannitol Salt Phenol Red Agar); Gram stain, catalase test, coagulase test, clumping factor A test, and Voges-Proskauer test. Confirmatory identification of *Staphylococcus* species: MALDI-TOF MS Technology and Bruker Daltonik MALDI Biotyper Classification Results (BCR) with average score value equal to 2.1718 ±0.2048 (maximum value equal to 2.477, minimum value equal to 1.706 and median value equal to 2.224). ∅: Sampling not performed during the study period (≥5 and ≤183 days). Types of previous surgical rehabilitation: Che (Cheiloplasty), Pal (Palatoplasty) and Rhi (Rhinoseptoplasty).
