# Supplementary material for: Oral Staphylococcus Species and MRSA Strains in Patients with Orofacial Clefts Undergoing Surgical Rehabilitation Diagnosed by MALDI-TOF MS
Source: Pathogens. 2024 Sep 5;13(9):763. doi: 10.3390/pathogens13090763 (PMC11434827; doi:10.3390/pathogens13090763)
Supplement: Supplementary file 1 [file pathogens-13-00763-s001.zip › Table S2.pdf]

**Supplementary Material – Table S2.** Dynamics and incidence of oral colonization by *Staphylococcus* species isolated from patients with orofacial clefts throughout the pre- and post-surgical periods (period A: admission to surgical ward prior to asepsis; period B: prior to surgical procedure and immediately after asepsis with PVP-I or chlorhexidine; period C: immediately after surgical rehabilitation; and period D: at the first patient return to the *Centro Pró-Sorriso aos Portadores de Fissuras Labial e Palatina*, Alfenas, MG, Brazil).

| **Patient code** | **Period A** | | | | | | | | | | **Period B** | | | | | | | | | |
|---|---|---|---|---|---|---|---|---|---|---|---|---|---|---|---|---|---|---|---|---|
| | **Bacterial species** | **I** | **II** | **III** | **Σ** | **∅** | **AM** | **SE** | **SN** | **SM** | **Bacterial species** | **I** | **II** | **III** | **Σ** | **∅** | **AM** | **SE** | **SN** | **SM** |
| **No surgical asepsis** | | | | | | | | | | | **Surgical asepsis using chlorhexidine** | | | | | | | | | |
| CLP6 | - | 0 | 0 | 0 | 0 | 1 | 1 | 0 | 0 | 0 | - | 0 | 0 | 0 | 0 | 1 | 1 | 0 | 0 | 0 |
| CLP10 | - | 0 | 0 | 0 | 0 | 1 | 1 | 0 | 0 | 0 | - | 0 | 0 | 0 | 0 | 1 | 1 | 0 | 0 | 0 |
| CLP13 | - | 0 | 0 | 0 | 0 | 1 | 1 | 0 | 0 | 0 | - | 0 | 0 | 0 | 0 | 1 | 1 | 0 | 0 | 0 |
| CLP14 | - | 0 | 0 | 0 | 0 | 1 | 1 | 0 | 0 | 0 | - | 0 | 0 | 0 | 0 | 1 | 1 | 0 | 0 | 0 |
| CLP16 | - | 0 | 0 | 0 | 0 | 1 | 1 | 0 | 0 | 0 | *S. saprophyticus* | 0 | 0 | 1 | 1 | 0 | 0 | 0 | 1 | 0 |
| CLP17 | - | 0 | 0 | 0 | 0 | 1 | 1 | 0 | 0 | 0 | - | 0 | 0 | 0 | 0 | 1 | 1 | 0 | 0 | 0 |
| CLP18 | - | 0 | 0 | 0 | 0 | 1 | 1 | 0 | 0 | 0 | - | 0 | 0 | 0 | 0 | 1 | 1 | 0 | 0 | 0 |
| CLP19 | - | 0 | 0 | 0 | 0 | 1 | 1 | 0 | 0 | 0 | - | 0 | 0 | 0 | 0 | 1 | 1 | 0 | 0 | 0 |
| CLP21 | - | 0 | 0 | 0 | 0 | 1 | 1 | 0 | 0 | 0 | - | 0 | 0 | 0 | 0 | 1 | 1 | 0 | 0 | 0 |
| CLP22 | - | 0 | 0 | 0 | 0 | 1 | 1 | 0 | 0 | 0 | - | 0 | 0 | 0 | 0 | 1 | 1 | 0 | 0 | 0 |
| CLP23 | *S. aureus* | 1 | 0 | 0 | 1 | 0 | 0 | 0 | 0 | 1 | - | 0 | 0 | 0 | 0 | 1 | 0 | 1 | 0 | 0 |
| CLP24 | - | 0 | 0 | 0 | 0 | 1 | 1 | 0 | 0 | 0 | - | 0 | 0 | 0 | 0 | 1 | 1 | 0 | 0 | 0 |
| CLP25 | - | 0 | 0 | 0 | 0 | 1 | 1 | 0 | 0 | 0 | - | 0 | 0 | 0 | 0 | 1 | 1 | 0 | 0 | 0 |
| CLP26 | - | 0 | 0 | 0 | 0 | 1 | 1 | 0 | 0 | 0 | - | 0 | 0 | 0 | 0 | 1 | 1 | 0 | 0 | 0 |
| CLP27 | *S. sciuri* | 0 | 0 | 1 | 1 | 0 | 0 | 0 | 0 | 1 | - | 0 | 0 | 0 | 0 | 1 | 0 | 1 | 0 | 0 |
| CLP28 | - | 0 | 0 | 0 | 0 | 1 | 1 | 0 | 0 | 0 | - | 0 | 0 | 0 | 0 | 1 | 1 | 0 | 0 | 0 |
| CLP29 | *S. epidermidis*, *S. saprophyticus* and *S. warneri* | 0 | 0 | 1 | 1 | 0 | 0 | 0 | 0 | 1 | - | 0 | 0 | 0 | 0 | 1 | 0 | 1 | 0 | 0 |
| CLP30 | *S. aureus* and *S. saprophyticus* | 0 | 1 | 0 | 1 | 0 | 0 | 0 | 0 | 1 | - | 0 | 0 | 0 | 0 | 1 | 0 | 1 | 0 | 0 |
| CLP31 | - | 0 | 0 | 0 | 0 | 1 | 1 | 0 | 0 | 0 | - | 0 | 0 | 0 | 0 | 1 | 1 | 0 | 0 | 0 |
| CLP32 | - | 0 | 0 | 0 | 0 | 1 | 1 | 0 | 0 | 0 | - | 0 | 0 | 0 | 0 | 1 | 1 | 0 | 0 | 0 |
| CLP33 | - | 0 | 0 | 0 | 0 | 1 | 1 | 0 | 0 | 0 | *S. saprophyticus* | 0 | 0 | 1 | 1 | 0 | 0 | 0 | 1 | 0 |
| CLP34 | - | 0 | 0 | 0 | 0 | 1 | 1 | 0 | 0 | 0 | - | 0 | 0 | 0 | 0 | 1 | 1 | 0 | 0 | 0 |
| CLP35 | - | 0 | 0 | 0 | 0 | 1 | 1 | 0 | 0 | 0 | - | 0 | 0 | 0 | 0 | 1 | 1 | 0 | 0 | 0 |
| CLP36 | *S. aureus* | 1 | 0 | 0 | 1 | 0 | 0 | 0 | 0 | 1 | - | 0 | 0 | 0 | 0 | 1 | 0 | 1 | 0 | 0 |
| CLP37 | *S. aureus* | 1 | 0 | 0 | 1 | 0 | 0 | 0 | 0 | 1 | - | 0 | 0 | 0 | 0 | 1 | 0 | 1 | 0 | 0 |
| CLP38 | - | 0 | 0 | 0 | 0 | 1 | 1 | 0 | 0 | 0 | - | 0 | 0 | 0 | 0 | 1 | 1 | 0 | 0 | 0 |
| CLP39 | - | 0 | 0 | 0 | 0 | 1 | 1 | 0 | 0 | 0 | - | 0 | 0 | 0 | 0 | 1 | 1 | 0 | 0 | 0 |
| CLP40 | *S. aureus* | 1 | 0 | 0 | 1 | 0 | 0 | 0 | 0 | 1 | - | 0 | 0 | 0 | 0 | 1 | 0 | 1 | 0 | 0 |
| CLP41 | - | 0 | 0 | 0 | 0 | 1 | 1 | 0 | 0 | 0 | - | 0 | 0 | 0 | 0 | 1 | 1 | 0 | 0 | 0 |
| CLP42 | - | 0 | 0 | 0 | 0 | 1 | 1 | 0 | 0 | 0 | - | 0 | 0 | 0 | 0 | 1 | 1 | 0 | 0 | 0 |
| CLP43 | *S. epidermidis* | 0 | 0 | 1 | 1 | 0 | 0 | 0 | 0 | 1 | - | 0 | 0 | 0 | 0 | 1 | 0 | 1 | 0 | 0 |
| CLP44 | *S. epidermidis* | 0 | 0 | 1 | 1 | 0 | 0 | 0 | 0 | 1 | - | 0 | 0 | 0 | 0 | 1 | 0 | 1 | 0 | 0 |
| CLP45 | - | 0 | 0 | 0 | 0 | 1 | 1 | 0 | 0 | 0 | - | 0 | 0 | 0 | 0 | 1 | 1 | 0 | 0 | 0 |
| CLP46 | - | 0 | 0 | 0 | 0 | 1 | 1 | 0 | 0 | 0 | - | 0 | 0 | 0 | 0 | 1 | 1 | 0 | 0 | 0 |
| CLP47 | - | 0 | 0 | 0 | 0 | 1 | 1 | 0 | 0 | 0 | - | 0 | 0 | 0 | 0 | 1 | 1 | 0 | 0 | 0 |
| CLP48 | *S. aureus* | 1 | 0 | 0 | 1 | 0 | 0 | 0 | 0 | 1 | - | 0 | 0 | 0 | 0 | 1 | 0 | 1 | 0 | 0 |
| CLP49 | *S. epidermidis* | 0 | 0 | 1 | 1 | 0 | 0 | 0 | 0 | 1 | - | 0 | 0 | 0 | 0 | 1 | 0 | 1 | 0 | 0 |
| CLP50 | *S. aureus* | 1 | 0 | 0 | 1 | 0 | 0 | 0 | 0 | 1 | - | 0 | 0 | 0 | 0 | 1 | 0 | 1 | 0 | 0 |
| CLP51 | *S. aureus* | 1 | 0 | 0 | 1 | 0 | 0 | 0 | 0 | 1 | - | 0 | 0 | 0 | 0 | 1 | 0 | 1 | 0 | 0 |
| CLP52 | - | 0 | 0 | 0 | 0 | 1 | 1 | 0 | 0 | 0 | - | 0 | 0 | 0 | 0 | 1 | 1 | 0 | 0 | 0 |
| CLP54 | *S. arlettae* and *S. saprophyticus* | 0 | 0 | 1 | 1 | 0 | 0 | 0 | 0 | 1 | - | 0 | 0 | 0 | 0 | 1 | 0 | 1 | 0 | 0 |
| CLP55 | - | 0 | 0 | 0 | 0 | 1 | 1 | 0 | 0 | 0 | - | 0 | 0 | 0 | 0 | 1 | 1 | 0 | 0 | 0 |
| CLP56 | *S. aureus* | 1 | 0 | 0 | 1 | 0 | 0 | 0 | 0 | 1 | - | 0 | 0 | 0 | 0 | 1 | 0 | 1 | 0 | 0 |
| CLP57 | - | 0 | 0 | 0 | 0 | 1 | 1 | 0 | 0 | 0 | - | 0 | 0 | 0 | 0 | 1 | 1 | 0 | 0 | 0 |
| CLP58 | *S. aureus* | 1 | 0 | 0 | 1 | 0 | 0 | 0 | 0 | 1 | - | 0 | 0 | 0 | 0 | 1 | 0 | 1 | 0 | 0 |
| CLP59 | *S. aureus* and *S. haemolyticus* | 0 | 1 | 0 | 1 | 0 | 0 | 0 | 0 | 1 | - | 0 | 0 | 0 | 0 | 1 | 0 | 1 | 0 | 0 |

| | | | | | | | | | | | | | | | | | | | | |
|---|---|---|---|---|---|---|---|---|---|---|---|---|---|---|---|---|---|---|---|---|
| | Σ (*n*) | 9 | 2 | 6 | 17 | 29 | 29 | 0 | 0 | 17 | Σ (*n*) | 0 | 0 | 2 | 2 | 44 | 27 | 17 | 2 | 0 |
| | Σ (%) | 19.6 | 4.3 | 13.0 | 37.0 | 63.0 | 63.0 | 0.0 | 0.0 | 37.0 | Σ (%) | 0.0 | 0.0 | 4.3 | 4.3 | 95.7 | 58.7 | 37.0 | 4.3 | 0.0 |
| **No surgical asepsis** | | | | | | | | | | | **Surgical asepsis using polyvinylpyrrolidone (PVPI)** | | | | | | | | | |
| CLP1 | *S. aureus* and *S. haemolyticus* | 0 | 1 | 0 | 1 | 0 | 0 | 0 | 0 | 1 | - | 0 | 0 | 0 | 0 | 1 | 0 | 1 | 0 | 0 |
| CLP2 | - | 0 | 0 | 0 | 0 | 1 | 1 | 0 | 0 | 0 | - | 0 | 0 | 0 | 0 | 1 | 1 | 0 | 0 | 0 |
| CLP3 | - | 0 | 0 | 0 | 0 | 1 | 1 | 0 | 0 | 0 | - | 0 | 0 | 0 | 0 | 1 | 1 | 0 | 0 | 0 |
| CLP4 | *S. aureus* and *S. sciuri* | 0 | 1 | 0 | 1 | 0 | 0 | 0 | 0 | 1 | - | 0 | 0 | 0 | 0 | 1 | 0 | 1 | 0 | 0 |
| CLP5 | - | 0 | 0 | 0 | 0 | 1 | 1 | 0 | 0 | 0 | - | 0 | 0 | 0 | 0 | 1 | 1 | 0 | 0 | 0 |
| CLP7 | *S. aureus* | 1 | 0 | 0 | 1 | 0 | 0 | 0 | 0 | 1 | - | 0 | 0 | 0 | 0 | 1 | 0 | 1 | 0 | 0 |
| CLP8 | *S. aureus* | 1 | 0 | 0 | 1 | 0 | 0 | 0 | 0 | 1 | - | 0 | 0 | 0 | 0 | 1 | 0 | 1 | 0 | 0 |
| CLP9 | *S. aureus* | 1 | 0 | 0 | 1 | 0 | 0 | 0 | 0 | 1 | - | 0 | 0 | 0 | 0 | 1 | 0 | 1 | 0 | 0 |
| CLP11 | - | 0 | 0 | 0 | 0 | 1 | 1 | 0 | 0 | 0 | - | 0 | 0 | 0 | 0 | 1 | 1 | 0 | 0 | 0 |
| CLP12 | - | 0 | 0 | 0 | 0 | 1 | 1 | 0 | 0 | 0 | - | 0 | 0 | 0 | 0 | 1 | 1 | 0 | 0 | 0 |
| CLP15 | - | 0 | 0 | 0 | 0 | 1 | 1 | 0 | 0 | 0 | - | 0 | 0 | 0 | 0 | 1 | 1 | 0 | 0 | 0 |
| CLP20 | *S. saprophyticus* | 0 | 0 | 1 | 1 | 0 | 0 | 0 | 0 | 1 | - | 0 | 0 | 0 | 0 | 1 | 0 | 1 | 0 | 0 |
| CLP53 | - | 0 | 0 | 0 | 0 | 1 | 1 | 0 | 0 | 0 | - | 0 | 0 | 0 | 0 | 1 | 1 | 0 | 0 | 0 |
| | Σ (*n*) | 3 | 2 | 1 | 6 | 7 | 7 | 0 | 0 | 6 | Σ (*n*) | 0 | 0 | 0 | 0 | 13 | 7 | 6 | 0 | 0 |
| | Σ (%) | 23.1 | 15.4 | 7.7 | 46.2 | 53.8 | 53.8 | 0.0 | 0.0 | 46.2 | Σ (%) | 0.0 | 0.0 | 0.0 | 0.0 | 100 | 53.8 | 46.2 | 0.0 | 0.0 |
| **No surgical asepsis** | | | | | | | | | | | **Surgical asepsis using chlorhexidine and PVPI** | | | | | | | | | |
| | Σ (*n*) | 12 | 4 | 7 | 23 | 36 | 36 | 0 | 0 | 23 | Σ (*n*) | 0 | 0 | 2 | 2 | 57 | 34 | 23 | 2 | 0 |
| | Σ (%) | 20.3 | 6.8 | 11.9 | 39.0 | 61.0 | 61.0 | 0.0 | 0.0 | 39.0 | Σ (%) | 0.0 | 0.0 | 3.4 | 3.4 | 96.6 | 57.6 | 39.0 | 3.4 | 0.0 |

I (*S. aureus*), II (*S. aureus* and *Staphylococcus* species), III (non-*aureus Staphylococcus* species), Σ (Total of presence of *Staphylococcus* species), ∅ (Absence of *Staphylococcus* species), AM (aseptic maintenance), SE (septic elimination), SN (septic neocolonization), SM (septic maintenance), — (oral sampling not performed during the study period: ≥5 and ≤183 days), 1 (present characteristic) and 0 (absent characteristic). Isolation and presumptive identification of *Staphylococcus* species: MSA selective culture medium (Mannitol Salt Phenol Red Agar); Gram stain, catalase test, coagulase test, clumping factor A test, and Voges-Proskauer test. Confirmatory identification of *Staphylococcus* species: MALDI-TOF MS Technology and Bruker Daltonik MALDI Biotyper Classification Results (BCR) with average score value equal to 2.1718 ±0.2048 (maximum value equal to 2.477, minimum value equal to 1.706 and median value equal to 2.224).

**Supplementary Material – Table S2.** Continued.

| Patient code | Period C Bacterial species | I | II | III | Σ | ∅ | AM | SE | SN | SM | SE+SN | Period D Bacterial species | I | II | III | Σ | ∅ | AM | SE | SN | SM | SE+SM |
|---|---|---|---|---|---|---|---|---|---|---|---|---|---|---|---|---|---|---|---|---|---|---|
| **Group chlorhexidine** | | | | | | | | | | | | **Group chlorhexidine** | | | | | | | | | | |
| CLP6 | - | 0 | 0 | 0 | 0 | 1 | 1 | 0 | 0 | 0 | 0 | - | 0 | 0 | 0 | 0 | 1 | 1 | 0 | 0 | 0 | 0 |
| CLP10 | *S. aureus* and *S. epidermidis* | 0 | 1 | 0 | 1 | 0 | 0 | 0 | 1 | 0 | 0 | - | 0 | 0 | 0 | 0 | 1 | 1 | 0 | 0 | 0 | 0 |
| CLP13 | - | 0 | 0 | 0 | 0 | 1 | 1 | 0 | 0 | 0 | 0 | — | — | — | — | — | — | — | — | — | — | — |
| CLP14 | - | 0 | 0 | 0 | 0 | 1 | 1 | 0 | 0 | 0 | 0 | — | — | — | — | — | — | — | — | — | — | — |
| CLP16 | - | 0 | 0 | 0 | 0 | 1 | 1 | 0 | 0 | 0 | 0 | *S. saprophyticus* | 0 | 0 | 1 | 1 | 0 | 0 | 0 | 1 | 0 | 0 |
| CLP17 | - | 0 | 0 | 0 | 0 | 1 | 1 | 0 | 0 | 0 | 0 | — | — | — | — | — | — | — | — | — | — | — |
| CLP18 | *S. aureus* | 1 | 0 | 0 | 1 | 0 | 0 | 0 | 1 | 0 | 0 | - | 0 | 0 | 0 | 0 | 1 | 1 | 0 | 0 | 0 | 0 |
| CLP19 | - | 0 | 0 | 0 | 0 | 1 | 1 | 0 | 0 | 0 | 0 | - | 0 | 0 | 0 | 0 | 1 | 1 | 0 | 0 | 0 | 0 |
| CLP21 | - | 0 | 0 | 0 | 0 | 1 | 1 | 0 | 0 | 0 | 0 | - | 0 | 0 | 0 | 0 | 1 | 1 | 0 | 0 | 0 | 0 |
| CLP22 | - | 0 | 0 | 0 | 0 | 1 | 1 | 0 | 0 | 0 | 0 | - | 0 | 0 | 0 | 0 | 1 | 1 | 0 | 0 | 0 | 0 |
| CLP23 | *S. epidermidis* | 0 | 0 | 1 | 1 | 0 | 0 | 0 | 0 | 0 | 1 | — | — | — | — | — | — | — | — | — | — | — |
| CLP24 | *S. aureus* and *S. epidermidis* | 0 | 1 | 0 | 1 | 0 | 0 | 0 | 1 | 0 | 0 | - | 0 | 0 | 0 | 0 | 1 | 1 | 0 | 0 | 0 | 0 |
| CLP25 | *S. aureus* | 1 | 0 | 0 | 1 | 0 | 0 | 0 | 1 | 0 | 0 | *S. aureus* | 1 | 0 | 0 | 1 | 0 | 0 | 0 | 1 | 0 | 0 |
| CLP26 | - | 0 | 0 | 0 | 0 | 1 | 1 | 0 | 0 | 0 | 0 | - | 0 | 0 | 0 | 0 | 1 | 1 | 0 | 0 | 0 | 0 |
| CLP27 | - | 0 | 0 | 0 | 0 | 1 | 0 | 1 | 0 | 0 | 0 | - | 0 | 0 | 0 | 0 | 1 | 0 | 1 | 0 | 0 | 0 |
| CLP28 | - | 0 | 0 | 0 | 0 | 1 | 1 | 0 | 0 | 0 | 0 | - | 0 | 0 | 0 | 0 | 1 | 1 | 0 | 0 | 0 | 0 |
| CLP29 | - | 0 | 0 | 0 | 0 | 1 | 0 | 1 | 0 | 0 | 0 | - | 0 | 0 | 0 | 0 | 1 | 0 | 1 | 0 | 0 | 0 |
| CLP30 | - | 0 | 0 | 0 | 0 | 1 | 0 | 1 | 0 | 0 | 0 | — | — | — | — | — | — | — | — | — | — | — |
| CLP31 | - | 0 | 0 | 0 | 0 | 1 | 1 | 0 | 0 | 0 | 0 | — | — | — | — | — | — | — | — | — | — | — |
| CLP32 | - | 0 | 0 | 0 | 0 | 1 | 1 | 0 | 0 | 0 | 0 | - | 0 | 0 | 0 | 0 | 1 | 1 | 0 | 0 | 0 | 0 |
| CLP33 | - | 0 | 0 | 0 | 0 | 1 | 1 | 0 | 0 | 0 | 0 | — | — | — | — | — | — | — | — | — | — | — |
| CLP34 | - | 0 | 0 | 0 | 0 | 1 | 1 | 0 | 0 | 0 | 0 | — | — | — | — | — | — | — | — | — | — | — |
| CLP35 | - | 0 | 0 | 0 | 0 | 1 | 1 | 0 | 0 | 0 | 0 | - | 0 | 0 | 0 | 0 | 1 | 1 | 0 | 0 | 0 | 0 |
| CLP36 | - | 0 | 0 | 0 | 0 | 1 | 0 | 1 | 0 | 0 | 0 | - | 0 | 0 | 0 | 0 | 1 | 0 | 1 | 0 | 0 | 0 |
| CLP37 | *S. aureus* | 1 | 0 | 0 | 1 | 0 | 0 | 0 | 0 | 1 | 0 | - | 0 | 0 | 0 | 0 | 1 | 0 | 1 | 0 | 0 | 0 |
| CLP38 | - | 0 | 0 | 0 | 0 | 1 | 1 | 0 | 0 | 0 | 0 | — | — | — | — | — | — | — | — | — | — | — |
| CLP39 | - | 0 | 0 | 0 | 0 | 1 | 1 | 0 | 0 | 0 | 0 | — | — | — | — | — | — | — | — | — | — | — |
| CLP40 | - | 0 | 0 | 0 | 0 | 1 | 0 | 1 | 0 | 0 | 0 | - | 0 | 0 | 0 | 0 | 1 | 0 | 1 | 0 | 0 | 0 |
| CLP41 | - | 0 | 0 | 0 | 0 | 1 | 1 | 0 | 0 | 0 | 0 | — | — | — | — | — | — | — | — | — | — | — |
| CLP42 | - | 0 | 0 | 0 | 0 | 1 | 1 | 0 | 0 | 0 | 0 | — | — | — | — | — | — | — | — | — | — | — |
| CLP43 | - | 0 | 0 | 0 | 0 | 1 | 0 | 1 | 0 | 0 | 0 | — | — | — | — | — | — | — | — | — | — | — |
| CLP44 | - | 0 | 0 | 0 | 0 | 1 | 0 | 1 | 0 | 0 | 0 | - | 0 | 0 | 0 | 0 | 1 | 0 | 1 | 0 | 0 | 0 |
| CLP45 | *S. lentus* | 0 | 0 | 1 | 1 | 0 | 0 | 0 | 1 | 0 | 0 | - | 0 | 0 | 0 | 0 | 1 | 1 | 0 | 0 | 0 | 0 |
| CLP46 | - | 0 | 0 | 0 | 0 | 1 | 1 | 0 | 0 | 0 | 0 | - | 0 | 0 | 0 | 0 | 1 | 1 | 0 | 0 | 0 | 0 |
| CLP47 | - | 0 | 0 | 0 | 0 | 1 | 1 | 0 | 0 | 0 | 0 | - | 0 | 0 | 0 | 0 | 1 | 1 | 0 | 0 | 0 | 0 |
| CLP48 | - | 0 | 0 | 0 | 0 | 1 | 0 | 1 | 0 | 0 | 0 | - | 0 | 0 | 0 | 0 | 1 | 0 | 1 | 0 | 0 | 0 |
| CLP49 | - | 0 | 0 | 0 | 0 | 1 | 0 | 1 | 0 | 0 | 0 | - | 0 | 0 | 0 | 0 | 1 | 0 | 1 | 0 | 0 | 0 |
| CLP50 | - | 0 | 0 | 0 | 0 | 1 | 0 | 1 | 0 | 0 | 0 | - | 0 | 0 | 0 | 0 | 1 | 0 | 1 | 0 | 0 | 0 |
| CLP51 | - | 0 | 0 | 0 | 0 | 1 | 0 | 1 | 0 | 0 | 0 | - | 0 | 0 | 0 | 0 | 1 | 0 | 1 | 0 | 0 | 0 |
| CLP52 | - | 0 | 0 | 0 | 0 | 1 | 1 | 0 | 0 | 0 | 0 | — | — | — | — | — | — | — | — | — | — | — |
| CLP54 | *S. aureus* | 1 | 0 | 0 | 1 | 0 | 0 | 0 | 0 | 0 | 1 | — | — | — | — | — | — | — | — | — | — | — |
| CLP55 | - | 0 | 0 | 0 | 0 | 1 | 1 | 0 | 0 | 0 | 0 | *S. aureus* | 1 | 0 | 0 | 1 | 0 | 0 | 0 | 1 | 0 | 0 |
| CLP56 | - | 0 | 0 | 0 | 0 | 1 | 0 | 1 | 0 | 0 | 0 | - | 0 | 0 | 0 | 0 | 1 | 0 | 1 | 0 | 0 | 0 |
| CLP57 | - | 0 | 0 | 0 | 0 | 1 | 1 | 0 | 0 | 0 | 0 | *S. aureus* | 1 | 0 | 0 | 1 | 0 | 0 | 0 | 1 | 0 | 0 |
| CLP58 | - | 0 | 0 | 0 | 0 | 1 | 0 | 1 | 0 | 0 | 0 | - | 0 | 0 | 0 | 0 | 1 | 0 | 1 | 0 | 0 | 0 |
| CLP59 | - | 0 | 0 | 0 | 0 | 1 | 0 | 1 | 0 | 0 | 0 | — | — | — | — | — | — | — | — | — | — | — |
| | Σ (*n*) | 4 | 2 | 2 | 8 | 38 | 24 | 14 | 5 | 1 | 2 | Σ (*n*) | 3 | 0 | 1 | 4 | 26 | 14 | 12 | 4 | 0 | 0 |
| | Σ (%) | 8.7 | 4.3 | 4.3 | 17.4 | 82.6 | 52.2 | 30.4 | 10.9 | 2.2 | 4.3 | Σ (%) | 10.0 | 0.0 | 3.3 | 13.3 | 86.7 | 46.7 | 40.0 | 13.3 | 0.0 | 0.0 |
| **Group polyvinylpyrrolidone (PVPI)** | | | | | | | | | | | | **Group polyvinylpyrrolidone (PVPI)** | | | | | | | | | | |
| CLP1 | - | 0 | 0 | 0 | 0 | 1 | 0 | 1 | 0 | 0 | 0 | - | 0 | 0 | 0 | 0 | 1 | 0 | 1 | 0 | 0 | 0 |
| CLP2 | - | 0 | 0 | 0 | 0 | 1 | 1 | 0 | 0 | 0 | 0 | - | 0 | 0 | 0 | 0 | 1 | 1 | 0 | 0 | 0 | 0 |
| CLP3 | - | 0 | 0 | 0 | 0 | 1 | 1 | 0 | 0 | 0 | 0 | - | 0 | 0 | 0 | 0 | 1 | 1 | 0 | 0 | 0 | 0 |
| CLP4 | - | 0 | 0 | 0 | 0 | 1 | 0 | 1 | 0 | 0 | 0 | *S. aureus* | 1 | 0 | 0 | 1 | 0 | 0 | 0 | 0 | 0 | 1 |
| CLP5 | - | 0 | 0 | 0 | 0 | 1 | 1 | | 0 | 0 | 0 | - | 0 | 0 | 0 | 0 | 1 | 1 | 0 | 0 | 0 | 0 |
| CLP7 | *S. aureus* | 1 | 0 | 0 | 1 | 0 | 0 | 0 | 0 | 1 | 0 | *S. aureus* | 1 | 0 | 0 | 1 | 0 | 0 | 0 | 0 | 1 | 0 |
| CLP8 | - | 0 | 0 | 0 | 0 | 1 | 0 | 1 | 0 | 0 | 0 | - | 0 | 0 | 0 | 0 | 1 | 0 | 1 | 0 | 0 | 0 |

| | | | | | | | | | | | | | | | | | | | | | |
|---|---|---|---|---|---|---|---|---|---|---|---|---|---|---|---|---|---|---|---|---|---|
| CLP9 | - | 0 | 0 | 0 | 0 | | 0 | | 0 | 0 | 0 | — | — | — | — | — | — | — | — | — | — | — |
| CLP11 | - | 0 | 0 | 0 | 0 | 1 | 1 | 0 | 0 | 0 | 0 | - | 0 | 0 | 0 | 0 | 1 | 1 | 0 | 0 | 0 | 0 |
| CLP12 | - | 0 | 0 | 0 | 0 | 1 | 1 | 0 | 0 | 0 | 0 | - | 0 | 0 | 0 | 0 | 1 | 1 | 0 | 0 | 0 | 0 |
| CLP15 | *S. aureus* | 1 | 0 | 0 | 1 | 0 | 0 | 0 | 1 | 0 | 0 | *S. aureus* | 1 | 0 | 0 | 1 | 0 | 0 | 0 | 1 | 0 | 0 |
| CLP20 | - | 0 | 0 | 0 | 0 | | 0 | | 0 | 0 | 0 | — | — | — | — | — | — | — | — | — | — | — |
| CLP53 | - | 0 | 0 | 0 | 0 | 1 | 1 | 0 | 0 | 0 | 0 | - | 0 | 0 | 0 | 0 | 1 | 1 | 0 | 0 | 0 | 0 |
| | Σ (*n*) | 2 | 0 | 0 | 2 | 11 | 6 | 5 | 1 | 1 | 0 | Σ (*n*) | 3 | 0 | 0 | 3 | 8 | 6 | 2 | 1 | 1 | 1 |
| | Σ (%) | 15.4 | 0.0 | 0.0 | 15.4 | 84.6 | 46.2 | 38.5 | 7.7 | 7.7 | 0.0 | Σ (%) | 27.3 | 0.0 | 0.0 | 27.3 | 72.7 | 54.5 | 18.2 | 9.1 | 9.1 | 9.1 |
| **Groups chlorhexidine and PVPI** | | | | | | | | | | | **Groups chlorhexidine and PVPI** | | | | | | | | | | |
| | Σ (*n*) | 6 | 2 | 2 | 10 | 49 | 30 | 19 | 6 | 2 | 2 | Σ (*n*) | 6 | 0 | 1 | 7 | 34 | 20 | 14 | 5 | 1 | 1 |
| | Σ (%) | 10.2 | 3.4 | 3.4 | 16.9 | 83.1 | 50.8 | 32.2 | 10.2 | 3.4 | 3.4 | Σ (%) | 14.6 | 0.0 | 2.4 | 17.1 | 82.9 | 48.8 | 34.1 | 12.2 | 2.4 | 2.4 |

I (*S. aureus*), II (*S. aureus* and *Staphylococcus* species), III (non-*aureus Staphylococcus* species), Σ (Total of presence of *Staphylococcus* species), ∅ (Absence of *Staphylococcus* species), AM (aseptic maintenance), SE (septic elimination), SN (septic neocolonization), SM (septic maintenance), — (oral sampling not performed during the study period: ≥5 and ≤183 days), 1 (present characteristic) and 0 (absent characteristic). Isolation and presumptive identification of *Staphylococcus* species: MSA selective culture medium (Mannitol Salt Phenol Red Agar); Gram stain, catalase test, coagulase test, clumping factor A test, and Voges-Proskauer test. Confirmatory identification of *Staphylococcus* species: MALDI-TOF MS Technology and Bruker Daltonik MALDI Biotyper Classification Results (BCR) with average score value equal to 2.1718 ±0.2048 (maximum value equal to 2.477, minimum value equal to 1.706 and median value equal to 2.224).
